# House Dust Mite Induces Distinct Cellular and Extracellular Vesicle-Associated miRNA Signatures in BEAS-2B Cells: Targeted Analysis of IL-4 Responses

**DOI:** 10.3390/cells15191744

**Published:** 2026-09-24

**Authors:** Isaac Kirubakaran Sundar, Allan Giri

**Affiliations:** Division of Pulmonary, Critical Care and Sleep Medicine, Department of Internal Medicine, University of Kansas Medical Center, Kansas City, KS 66160, USA; allangiri@yahoo.in

**Keywords:** bronchial epithelial cells, extracellular vesicles, house dust mite, miRNA signatures, allergic asthma, IL-4

## Abstract

**Highlights:**

- HDM exposure induced distinct cellular and extracellular vesicle (EV)-associated miRNA profiles in BEAS-2B cells, with limited overlap between compartments.
- HDM-responsive EV-associated miRNAs (miR-612, miR-302d-3p, miR-15a-5p, and miR-125b-5p) were validated, and most showed reduced expression following IL-4 stimulation.
- EV-associated miRNAs may contribute to epithelial cell communications during allergic airway inflammation. These miRNAs represent potential targets for future mechanistic studies and biomarker development in asthma.

**Abstract:**

Airway epithelial cells actively regulate allergic asthma through allergen sensing, type 2 cytokine responses, and extracellular vesicle (EV)-mediated intercellular communication. MicroRNAs (miRNAs) are important post-transcriptional regulators of epithelial inflammation and remodeling; however, the extent to which allergen exposure differentially alters cellular and EV-associated miRNA profiles remains incompletely understood. This study characterized miRNA responses to house dust mite (HDM) exposure in BEAS-2B bronchial epithelial cells and their EV-enriched fractions and subsequently examined selected HDM-responsive miRNAs following interleukin-4 (IL-4) stimulation. BEAS-2B cells were exposed to HDM extract or IL-4 for 72 h. EV-enriched preparations were isolated from conditioned media and characterized by transmission electron microscopy, nanoparticle tracking analysis, immunoblotting of EV-associated markers, and uptake assays. Global cellular and EV-associated miRNA profiling was performed in control and HDM-treated samples using the NanoString nCounter Human v3 miRNA assay, followed by qRT-PCR validation of selected candidates. HDM-responsive miRNAs were subsequently evaluated in IL-4-treated cells and corresponding EV-associated RNA by targeted qRT-PCR. Differentially expressed miRNAs were subjected to pathway-enrichment analysis. HDM exposure induced substantial remodeling of the cellular miRNA landscape, with 39 differentially expressed miRNAs identified in BEAS-2B cells. In contrast, EV-associated RNA exhibited a smaller but distinct set of differentially expressed miRNAs, including miR-612, miR-302d-3p, miR-125b-5p, and miR-15a-5p. Notably, miR-15a-5p displayed opposite directional changes in cellular and EV-associated RNA, highlighting compartment-specific differences in miRNA abundance. qRT-PCR independently supported several EV-associated candidates, including miR-612, miR-302d-3p, miR-15a-5p, and miR-125b-5p. Pathway analyses linked HDM-responsive miRNAs to cell-cycle regulation, p53 signaling, FoxO signaling, autophagy, and inflammatory pathways. Targeted qRT-PCR analysis revealed that many selected HDM-responsive miRNAs exhibited reduced expression following IL-4 stimulation compared with their respective untreated controls, indicating different directional responses for several candidates across the two stimulation paradigms . HDM exposure is associated with distinct cellular and EV-associated miRNA responses in the BEAS-2B bronchial epithelial model. The limited overlap between cellular and EV-associated miRNA profiles indicates that specific miRNAs are present at different levels in intracellular and EV compartments. In addition, several HDM-responsive miRNAs exhibited divergent responses following IL-4 stimulation, supporting context-dependent regulation by allergen and cytokine signaling. These findings identify EV-associated miRNAs as candidates for future mechanistic studies in the context of epithelial intercellular communication and allergic airway inflammation.

## 1. Introduction

The airway epithelium is now recognized as an active immune-sensing interface rather than merely a passive physical barrier. Continuously exposed to inhaled allergens, pollutants, and microbial products, the respiratory epithelium plays a central role in regulating airway immune responses [1]. Upon activation, epithelial cells can undergo barrier disruption and mucus hypersecretion, and release chemokines and epithelial alarmins such as thymic stromal lymphopoietin (TSLP), interleukin-33 (IL-33), and IL-25. These signals initiate and amplify type 2 airway inflammation by activating a range of immune and structural cells, including dendritic cells, type 2 innate lymphoid cells (ILC2s), Th2 cells, mast cells, eosinophils, and airway structural cells, thereby shaping downstream immune and remodeling responses [1,2,3,4,5].

House dust mite (HDM) is among the most clinically relevant aeroallergens in allergic asthma, as it contains proteases and pathogen-associated molecular patterns that can injure the epithelial barrier and activate innate immune pathways. Unlike stimulation with a single cytokine, HDM acts as a complex allergen-derived stimulus, simultaneously engaging multiple pathways such as epithelial barrier disruption, calcium signaling, Toll-like receptor activation, protease-activated receptors, oxidative stress responses, and alarmin release. HDM allergens have been shown to induce epithelial calcium signaling and alarmin production involving PAR-2, PAR-4, TRPV1, IL-33, and TSLP. Additionally, HDM-induced PAR-2/ERK signaling promotes TSLP secretion and airway remodeling through epithelial–fibroblast communication [6,7]. In contrast, type 2 cytokines such as IL-4 and IL-13 primarily signal through the IL-4 receptor and activate STAT6-dependent transcriptional programs that regulate IgE biology, goblet cell metaplasia, mucus production, periostin expression, and other features of T2-high asthma [8,9,10]. Examining the behavior of HDM allergen-responsive miRNAs under IL-4 stimulation may therefore provide insight into whether selected miRNA responses are shared between complex allergen exposure and canonical type 2 cytokine signaling.

Beyond intracellular miRNA activity, extracellular vesicles (EVs) have emerged as important mediators of airway intercellular communication. Airway epithelial-derived EVs transport miRNAs, mRNAs, proteins, lipids, and danger-associated molecular patterns that can influence neighboring epithelial cells as well as macrophages, dendritic cells, fibroblasts, and lymphocytes [3,11,12,13,14,15,16,17,18]. A recent systematic review highlighted that epithelial EVs can modulate IgE-mediated allergic sensitization, Th2 polarization, cytokine secretion, macrophage activation, and barrier dysfunction, and, in some contexts, promote immune tolerance through regulatory T-cell responses [19].

EV-associated miRNAs are of particular interest in asthma, as they may function both as biomarkers of disease activity and as mediators of intercellular signaling [20,21]. Studies using primary human bronchial epithelial cells have shown that EV miRNA profiles are altered during asthma development and following IL-13 exposure, with EV miRNA changes correlating with lung function in children [15]. Analyses of EVs isolated from plasma and bronchoalveolar lavage fluid of patients with severe asthma further support the association between EV miRNA signatures, disease severity, inflammation, and impaired lung function [22,23,24]. However, it remains unclear whether EV miRNA cargo simply reflects intracellular miRNA abundance or instead exhibits selective, stimulus-dependent enrichment within the extracellular vesicle compartment. Emerging evidence suggests that EV-associated miRNA cargo may be selectively regulated rather than simply reflecting intracellular miRNA abundance, raising the possibility that cellular and EV-associated miRNA profiles provide distinct biological information [25,26].

Despite growing interest in epithelial EV biology, relatively few studies have directly compared intracellular and EV-associated miRNA responses following allergen exposure, leaving the relationship between cellular and EV-associated miRNA abundance incompletely understood [15,16,27]. Specifically, it remains unclear whether EV-associated miRNA profiles primarily reflect intracellular expression patterns or represent a distinct compartment with differential miRNA enrichment.

Furthermore, it is not known whether allergen-responsive miRNAs exhibit similar regulation following type 2 cytokine stimulation. Since HDM and IL-4 activate overlapping but biologically distinct epithelial signaling pathways, comparing HDM-responsive miRNAs under IL-4 stimulation may provide insight into stimulus-dependent regulation of epithelial miRNA responses.

Therefore, the primary objective of this study was to characterize cellular and EV-associated miRNA responses to HDM exposure in the BEAS-2B bronchial epithelial cell model using global NanoString profiling, qRT-PCR validation and bioinformatic pathway analysis. As a secondary targeted analysis, selected HDM-responsive miRNAs were examined following IL-4 stimulation to assess whether they exhibited similar or divergent responses to a canonical type 2 cytokine stimulus. Given that BEAS-2B cells do not fully recapitulate the complexity of differentiated primary human airway epithelial cells, these findings are interpreted within the context of this experimental model and provide a foundation for future validation in primary airway epithelial systems.

## 2. Materials and Methods

### 2.1. BEAS-2B Cell Culture and Treatments

The immortalized human bronchial epithelial cell line BEAS-2B (ATCC, Manassas, VA, USA) was cultured in a 50:50 mixture of Dulbecco’s Modified Eagle Medium and Ham’s F-12 (DMEM/F-12; Mediatech, Manassas, VA, USA) supplemented with 10% extracellular vesicle (EV)-depleted fetal bovine serum (FBS One Shot; GibcoWaltham, MA, USA, Cat# A27208-03), 15 mM HEPES, penicillin (100 U/mL), and streptomycin (100 µg/mL). Cells were maintained at 37 °C in a humidified incubator with 5% CO_2_ and routinely grown to confluence in T-75 flasks.

For experimental treatments, BEAS-2B cells (4 × 10^6^ per plate) were seeded in 100-mm culture dishes and grown to approximately 80–90% confluence in DMEM/F-12 containing 1% EV-depleted FBS. Cells were then treated with house dust mite (HDM) extract (30 µg/mL) or the Th2 cytokine interleukin-4 (IL-4; 50 ng/mL) for 72 h under standard culture conditions. Untreated cells maintained under identical conditions served as controls.

At 72 h post-treatment, cells were harvested and stored at −80 °C for total RNA isolation. Conditioned media were collected simultaneously for isolation of EV-enriched preparations and downstream characterization. RNA isolated from control and HDM-treated BEAS-2B cells and corresponding EV-enriched preparations was used for NanoString miRNA profiling and qRT-PCR validation. RNA from IL-4-treated cells and corresponding EV-enriched preparations was used for targeted qRT-PCR assessment of selected HDM-responsive miRNAs.

### 2.2. Extracellular Vesicle Isolation from BEAS-2B Conditioned Media

Conditioned media collected from control and treated BEAS-2B cells were subjected to differential centrifugation to isolate EV-enriched fractions. Briefly, conditioned media were first centrifuged at 400× *g* for 10 min at 20 °C to remove cells, apoptotic bodies, and large debris. The supernatant was carefully transferred to clean tubes and centrifuged at 15,500× *g* for 30 min at 20 °C to pellet larger particles and vesicular materials; this pellet was retained as a microvesicle fraction when required.

The resulting supernatant was transferred to ultracentrifuge tubes and centrifuged at 100,000× *g* for 1 h at 20 °C to pellet small EVs. The EV pellet was washed with sterile 1× phosphate-buffered saline (PBS) and subjected to a second ultracentrifugation step at 100,000× *g* for 1 h at 20 °C to reduce contaminating proteins. The final EV pellet was resuspended in 200 µL of sterile 1× PBS.

Isolated EV-enriched preparations were used for particle size and concentration analysis using nanoparticle tracking analysis (NanoSight NS300, Malvern Panalytical, Worcestershire, UK) and transmission electron microscopy. EV preparations were also processed for total RNA isolation for downstream miRNA expression analyses.

### 2.3. Extracellular Vesicle Protein Characterization

EV preparations were further characterized by immunoblotting of EV-associated and intracellular protein markers in accordance with MISEV2023 recommendations [28]. EV preparations obtained from control and house dust mite (HDM)-treated BEAS-2B cells were lysed, and total protein was extracted. Protein profiles and loading consistency were initially assessed using stain-free gel electrophoresis.

Equal amounts of total EV protein were separated by sodium dodecyl sulfate-polyacrylamide gel electrophoresis (SDS-PAGE) and subsequently transferred onto nitrocellulose membranes. Membranes were blocked and incubated overnight at 4 °C with primary antibodies (1:1000 dilution) targeting the EV-associated markers CD63 (Santa Cruz Biotechnology [SCBT], Santa Cruz, CA, USA; sc-15363), TSG101 (Proteintech, Rosemont, IL, USA; 67381-1-Ig), and Syntenin-1 (SCBT; sc-515538). To assess potential contamination from intracellular components, membranes were also probed for the endoplasmic reticulum-associated proteins GRP94 (SCBT; sc-393402) and calnexin (SCBT; sc-11397).

Following primary antibody incubation, membranes were washed with Tris-buffered saline containing 0.1% Tween-20 (TBST) and incubated for 2 h at room temperature with horseradish peroxidase (HRP)-conjugated secondary antibodies (goat anti-mouse IgG [H + L] or goat anti-rabbit IgG [H + L]; Invitrogen, Carlsbad, CA, USA) diluted 1:10,000 in TBST supplemented with 5% bovine serum albumin (BSA). Immunoreactive bands were visualized using enhanced chemiluminescence (ECL; Thermo Fisher Scientific, Waltham, MA, USA) and imaged using a ChemiDoc Imaging System (Bio-Rad, Hercules, CA, USA).

When multiple protein targets were analyzed on the same membrane, blots were stripped using a commercial stripping buffer (Thermo Fisher Scientific), thoroughly washed, and reprobed with the appropriate primary and secondary antibodies. The presence of EV-enriched markers together with the absence or minimal detection of endoplasmic reticulum-associated proteins was used to assess the purity and identity of the EV preparations.

### 2.4. Transmission Electron Microscopy Analysis of BEAS-2B-Derived Extracellular Vesicles

EV-enriched preparations were visualized by transmission electron microscopy (TEM) as described previously [29,30]. Briefly, 2–4 µL of isolated EV suspension was fixed in 4% paraformaldehyde and applied onto carbon-coated copper grids. After adsorption, grids were washed twice with 1× phosphate-buffered saline (PBS), followed by a wash with 50 mM glycine in PBS for 3 min to quench residual aldehyde groups. Grids were then incubated in 0.5% bovine serum albumin (BSA) in PBS for 10 min to block nonspecific binding.

Following washing, EVs were negatively stained with 2% uranyl acetate, air-dried, and examined using a Hitachi 7650 Analytical transmission electron microscope (Tokyo, Japan). Images were acquired at appropriate magnifications to assess EV morphology and size distribution.

### 2.5. Nanoparticle Tracking Analysis (NanoSight NS300)

Particle size distribution and concentration of BEAS-2B-derived EV-enriched preparations were measured by nanoparticle tracking analysis (NTA) using the NanoSight NS300 system (Malvern Panalytical, Worcestershire, UK). EV preparations isolated by ultracentrifugation were diluted 1:1000 in particle-free phosphate-buffered saline (PBS), gently mixed, and introduced into the sample chamber using a syringe pump at a constant flow rate. For each sample, three 60-s video recordings were acquired.

Instrument settings, including camera gain, shutter speed, and manual focus, were optimized to ensure visualization of the maximal number of particles and were held constant across all samples. Post-acquisition analysis parameters were similarly standardized for all datasets to ensure consistency. Particle movement was tracked and analyzed using NanoSight software NTA 3.1. Build 3.1.46 (Malvern Panalytical, Worcestershire, UK) to calculate EV concentration and size distribution, as described previously [30,31].

### 2.6. Extracellular Vesicle Labeling and Confocal Microscopy

Extracellular vesicles (EVs) isolated from control BEAS-2B cells were fluorescently labeled using the ExoGlow™ membrane EV labeling kit (Cat# EXOGM600A-1; System Biosciences, Palo Alto, CA, USA) according to the manufacturer’s instructions. Briefly, EVs prepared using the differential ultracentrifugation protocol described above were quantified using a micro-BCA protein assay, and 50–100 µg of EVs was used for labeling.

For each reaction, 2 µL of ExoGlow labeling dye was added to 12 µL of reaction buffer in a 0.6-mL microcentrifuge tube and mixed thoroughly. The quantified EV preparation was then added to the labeling mixture and incubated at room temperature for 30 min with gentle mixing to allow membrane incorporation of the fluorescent dye. To remove excess unbound dye, the labeled EV suspension was purified using PD SpinTrap™ G-25 desalting columns (GE Healthcare, Chicago, IL, USA) following the manufacturer’s protocol.

The purified fluorescently labeled EVs were immediately used for uptake experiments in recipient BEAS-2B cells. Labeled EVs were incubated with BEAS-2B cells under standard culture conditions, and uptake was analyzed 24 h post-incubation. Fluorescent images were acquired using a laser scanning confocal microscope (Olympus FV1000, Tokyo, Japan) to visualize the cellular distribution of fluorescence associated with labeled EV preparations.

### 2.7. Extracellular Vesicle Total RNA Isolation

Total RNA was isolated from BEAS-2B cell-derived extracellular vesicles (EVs) using the Exosomal/EV RNA Isolation Kit (Norgen Biotek Corp., Thorold, ON, , Canada) according to the manufacturer’s instructions. Briefly, 150 µL of EV suspension resuspended in 1× PBS was used as the starting material. To each sample, 300 µL of Lysis Buffer A and 37.5 µL of Lysis Additive B were added, and the mixture was vortexed for 10 s followed by incubation at room temperature for 10 min to ensure complete lysis.

Subsequently, an appropriate volume of 96–100% ethanol was added, and the samples were vortexed again for 10 s. A total of 500 µL of the lysate–ethanol mixture was transferred to a mini spin column and centrifuged at 3300× *g* for 1 min. The flow-through was discarded, and the remaining sample was passed through the same column in a second centrifugation step. The column was then washed twice with 600 µL of Wash Solution A, and centrifuged at 3300× *g* for 30 s each time, and the flow-through discarded. To remove residual wash buffer, the column was centrifuged at 13,000× *g* for 1 min.

Following washing, the spin column was placed into a clean 1.5-mL microcentrifuge tube. EV RNA was eluted by adding 50 µL of Elution Solution A, incubating at room temperature for 2 min, and centrifuging at 400× *g* for 1 min, followed by a second centrifugation at 6200× *g* for 2 min. The eluted EV RNA was immediately used for downstream NanoString miRNA expression analysis and qRT-PCR validation.

### 2.8. Total RNA Isolation and cDNA Synthesis for miRNA Analysis

Total RNA from BEAS-2B cells was extracted using the miRNeasy Micro Kit (Qiagen, Valencia, CA, USA) following the manufacturer’s protocol. RNA concentration and purity were assessed using a NanoDrop spectrophotometer (Thermo Scientific, Wilmington, DE, USA).

Complementary DNA (cDNA) for miRNA analysis was synthesized using the miScript II RT Kit (Qiagen). For reverse transcription, 1 µg of total cellular RNA or 100 ng of EV RNA was used per reaction. RNA samples were adjusted to a final volume of 12 µL with RNase-free water, followed by addition of 4 µL of 5× miScript HiSpec Buffer, 2 µL of 10× miScript Nucleic Mix, and 2 µL of miScript Reverse Transcriptase Mix. Reaction mixtures were gently mixed, briefly centrifuged, and incubated in a thermal cycler under the following conditions: 60 min at 37 °C, followed by 5 min at 95 °C to inactivate the reverse transcriptase. The resulting cDNA was diluted as required for quantitative RT-PCR analysis and stored at −20 °C until use.

### 2.9. Quantitative Real-Time PCR (qRT-PCR) Analysis

Quantitative real-time PCR (qRT-PCR) was performed to independently assess selected miRNA expression changes identified by NanoString profiling. cDNA generated from total RNA or EV RNA was diluted 1:10 or 1:5 and used as a template for qRT-PCR. Amplification reactions were carried out using the miScript SYBR Green PCR Kit (Qiagen), which includes a universal reverse primer, on a CFX Opus 96 Real-Time PCR System (Bio-Rad).

miRNA-specific forward primers were designed using mature human miRNA sequences obtained from miRBase, and primers were synthesized by Integrated DNA Technologies (IDT; www.idtdna.com) (Table S1). Each reaction was performed according to the manufacturer’s instructions, and amplification specificity was verified by melt-curve analysis.

Relative miRNA expression levels were calculated using the 2^−ΔΔCt^ method. RNU6-1 was used as the endogenous reference control for total cellular miRNA, while RNA5S1 served as the reference gene for EV-associated miRNA analysis, as described previously [32]. All reactions were performed in technical replicates, and data are presented as fold change relative to control samples.

For the secondary IL-4 analysis, qRT-PCR was used to evaluate selected miRNAs identified from the HDM NanoString discovery dataset in cellular and EV-associated RNA obtained from IL-4-treated samples. This analysis was targeted and was not intended to represent comprehensive profiling of the IL-4-responsive miRNA landscape.

### 2.10. NanoString Human miRNA Assay and nSolver Data Analysis

Global miRNA expression profiling was performed on cellular and EV-associated RNA from control and HDM-treated samples using the NanoString nCounter Human v3 miRNA Expression Assay (Cat. # CSO-MIR3-12; NanoString Technologies, Seattle, WA, USA), which profiles 827 human miRNAs, 5 mRNAs, and 25 internal reference controls. For each sample, 300 ng of total cellular RNA or 100 ng of extracellular vesicle (EV) RNA, quantified by NanoDrop spectrophotometry, was hybridized and processed according to the manufacturer’s protocol using the NanoString nCounter Profiler system.

Raw miRNA counts were analyzed using nSolver™ Analysis Software version 4.0 (NanoString Technologies). For BEAS-2B total RNA samples (n = 6/group), data normalization was performed using a global mean normalization approach, in which the total counts of the 100 most highly expressed miRNAs across all samples were used as the normalization factor, as described previously [33].

For BEAS-2B EV-associated RNA samples, normalization was performed using a set of empirically identified stable miRNAs with average counts >100 and a low coefficient of variation (%CV). These included miR-100-5p, miR-130a-3p, miR-191-5p, miR-22-3p, miR-222-3p, miR-320e, and miR-4454 + miR-7975. This strategy was used to obtain normalized counts, fold-change ratios (HDM vs. control), nominal *p* values, and false discovery rate (FDR)-adjusted *p* values.

Only miRNAs with an average normalized count of ≥80 in at least one group within a given pairwise comparison were retained for downstream analysis. Differential expression among these miRNAs was defined by a nominal *p* value < 0.05. Fold-change values were reported to indicate the magnitude and direction of treatment-associated differences but were not used as an independent criterion for differential expression. Benjamini–Hochberg false discovery rate (FDR)-adjusted *p* values were also calculated and reported to account for multiple testing; however, they were not used as the primary criterion for candidate miRNA selection.

Given the high-dimensional nature of the dataset and sample size (n = 6/group), NanoString analyses were considered exploratory and used to identify treatment-associated miRNA candidates for subsequent qRT-PCR assessment. Candidate miRNAs were prioritized based on expression abundance, statistical significance, effect size, and subsequent qRT-PCR validation. Hierarchical clustering analysis was performed using Morpheus v5.3.1. (Broad Institute; https://software.broadinstitute.org/morpheus/ (accessed on 27 July 2021)), based on normalized NanoString counts. Clustering parameters were set to one minus Pearson correlation as the distance metric, average linkage, and clustering of both rows (miRNAs) and columns (samples).

### 2.11. miRNA Database and Bioinformatic Analyses

Venn diagrams were generated using an online tool (https://bioinformatics.psb.ugent.be/webtools/Venn/ (accessed on 27 July 2021)) to compare differentially expressed (DE) miRNAs identified in HDM-treated versus control BEAS-2B cells, distinguishing miRNAs that were shared or unique between total cellular RNA and extracellular vesicle (EV) RNA datasets.

To identify biological pathways potentially regulated by HDM-induced miRNA changes, all DE miRNAs from both total RNA and EV RNA were analyzed using the DIANA miRPath v4.0 online platform (accessed on 27 July 2021) for Kyoto Encyclopedia of Genes and Genomes (KEGG) pathway enrichment analysis [34]. An miRNA-centric analysis strategy was employed using the following parameters: TarBase v8.0 as the target database, direct TarBase-validated targets, organism set to Homo sapiens, and miRNA annotation version miRBase v22.2 [35,36]. Pathway enrichment was performed using the pathway union (merging) method, with the classic enrichment analysis option selected, a *p*-value threshold of 0.05, and false discovery rate (FDR) correction applied.

KEGG pathway analyses were conducted separately for upregulated and downregulated DE miRNAs identified in BEAS-2B total RNA and EV RNA to identify enriched biological pathways associated with DE miRNAs.

### 2.12. miRNA Target Prediction

Experimentally validated target genes of the differentially expressed miRNAs were identified using miRTarBase v9.0 [37]. To ensure high confidence in miRNA-target associations, only interactions supported by strong experimental validation methods, including reporter assays, Western blotting, and qRT-PCR, were retained. This analysis was performed for the differentially expressed miRNAs identified by NanoString profiling in HDM-treated BEAS-2B cells and BEAS-2B-derived EVs relative to their respective controls.

### 2.13. miRNA-mRNA Target Association Using GSE158752

To assess the potential clinical relevance of identified miRNAs, expression of selected validated target genes was examined in the publicly available bronchial epithelial transcriptomic dataset GSE158752 [38,39]. This dataset comprises bronchial epithelial cell transcriptomic profiles from 67 individuals, including 17 healthy controls (HC), 25 patients with mild-to-moderate asthma (M), and 25 patients with severe asthma (S). Bronchial epithelial cells were collected by endobronchial brushing and subjected to RNA sequencing [38,39]. Expression data for mRNA targets of differentially expressed miRNAs identified in our study were extracted from GSE158752 and compared across clinical groups to assess potential associations between miRNA dysregulation and asthma severity.

### 2.14. Statistical Analysis

Differential-expression analyses of NanoString data were performed as described above. Statistical analyses of qRT-PCR data were conducted using an unpaired two-tailed Student’s *t*-test. NTA measurements were analyzed by one-way ANOVA followed by Tukey’s multiple-comparisons test. Data are presented as mean ± SEM, and *p* < 0.05 was considered statistically significant. All analyses were conducted using GraphPad Prism version 9 (GraphPad Software, La Jolla, CA, USA).

## 3. Results

### 3.1. Isolation and Characterization of BEAS-2B-Derived Extracellular Vesicles

Previous studies have shown that airway epithelial cells release EVs under inflammatory conditions and that EV cargo may contribute to epithelial–immune communication in asthma [17]. Based on these observations, we examined whether stimulation with house dust mite (HDM) allergen or the Th2 cytokine IL-4 would alter the concentration and size distribution of particles recovered in EV-enriched preparations. EV-enriched fractions were isolated from conditioned media collected from control, HDM-treated, and IL-4-treated BEAS-2B cells using differential centrifugation followed by ultracentrifugation, as described in Section 2.

#### 3.1.1. Transmission Electron Microscopy (TEM)

Transmission electron microscopy (TEM) demonstrated vesicle-like particles with typical rounded or cup-shaped morphology in EV preparations isolated from control and HDM-treated BEAS-2B cells. TEM analysis was not performed on EVs derived from IL-4-treated samples (Figure 1A).

#### 3.1.2. Nanoparticle Tracking Analysis (NTA)

Nanoparticle tracking analysis (NTA; NanoSight NS300) was used to assess particle concentration and size distribution across all experimental groups. Relative to controls, particle concentrations were increased in preparations derived from HDM- and IL-4-treated cells, whereas mean and modal particle sizes remained comparable among groups. Representative NTA plots and summary data for EV concentration and size distribution are shown in Figure 1A,B.

#### 3.1.3. Immunoblotting Analysis of EV Markers

To provide complementary protein-based characterization, EV-enriched preparations were analyzed by stain-free gel electrophoresis and immunoblotting. Stain-free analysis demonstrated broad total protein profiles across the analyzed EV preparations (Figure S1A). Immunoblotting detected the EV-associated proteins TSG101, Syntenin-1, and CD63, including the expected heterogeneous glycosylated CD63 pattern. In contrast, the endoplasmic reticulum-associated proteins GRP94 and calnexin were not detected in the analyzed EV preparations (Figure S1B). Collectively, these characterization approaches confirmed the presence of EV-associated markers and the absence of detectable GRP94 and calnexin in the analyzed preparations.

#### 3.1.4. EV Labeling and Uptake

To assess whether BEAS-2B-derived EVs could be taken up by recipient epithelial cells, EVs isolated from control BEAS-2B cells were labeled using the ExoGlow™ membrane EV labeling kit and incubated with recipient BEAS-2B cells. Punctate and perinuclear-associated fluorescence was observed in recipient BEAS-2B cells after 24 h of incubation with fluorescently labeled EV preparations (Figure S2).

### 3.2. HDM Induces Distinct miRNA Profiles in BEAS-2B Cells

To assess the impact of HDM exposure on miRNA expression in the BEAS-2B bronchial epithelial cell model, we performed NanoString miRNA profiling on total RNA isolated from BEAS-2B cells treated with HDM for 72 h. Compared to control cells, HDM exposure resulted in significant differential expression of 39 miRNAs, with 15 upregulated and 24 downregulated (Figure 2A,B).

### 3.3. HDM Induces Distinct miRNA Profiles in BEAS-2B Extracellular Vesicles

To determine whether HDM-induced cellular miRNA changes were also reflected in extracellular vesicles, NanoString profiling was performed on EV-associated RNA isolated from the corresponding BEAS-2B-derived EV preparations. Compared with control EVs, HDM-treated EVs exhibited a smaller but distinct set of differentially expressed miRNAs. Specifically, six miRNAs (miR-612, miR-302d-3p, miR-873-3p, miR-1246, miR-15a-5p, and miR-125b-5p) met the prespecified differential-expression criteria as upregulated, whereas miR-494-3p and miR-21-5p met these criteria as downregulated (Figure 3A,B).

Hierarchical clustering of differentially expressed miRNAs separated HDM-treated and control samples in both total cellular RNA and EV-associated RNA datasets. Fold changes, nominal *p* values, and false-discovery-rate (FDR)-adjusted *p* values for all differentially expressed miRNAs are summarized in Figure 2B and Figure 3B. Normalized NanoString counts for upregulated and downregulated miRNAs are provided in Figures S3A,B and S4A,B. Overall, a greater number of differentially expressed miRNAs were identified in total cellular RNA than in EV-associated RNA following HDM exposure.

### 3.4. Comparative BEAS-2B Cellular and EV-Associated miRNA Analysis

To directly compare cellular and EV-associated miRNA responses, we performed Venn diagram analysis. Among the 39 differentially expressed miRNAs identified in total cellular RNA and the eight identified in EV-associated RNA, only one miRNA, miR-15a-5p, was shared between the two datasets (Figure S5A). Notably, miR-15a-5p exhibited opposite directional changes following HDM exposure, decreasing in cellular RNA while increasing in EV-associated RNA.

Unsupervised hierarchical clustering and principal component analysis (PCA) demonstrated clear separation of HDM-treated and control samples in both the cellular and EV-associated RNA datasets (Figure S5B). Fold changes, nominal *p* values, and FDR-adjusted *p* values for differentially expressed miRNAs are presented in Figure 2B and Figure 3B, whereas normalized expression counts are provided in Figures S3A,B and S4A,B.

To examine the potential biological processes associated with these miRNA changes, pathway enrichment analysis was performed using DIANA miRPath. Differentially expressed miRNAs identified in HDM-treated BEAS-2B cells were enriched in several KEGG pathways, including cell-cycle regulation, p53 signaling, FoxO signaling, cellular senescence, and autophagy (Figure 2C). Analysis of EV-associated miRNAs identified several overlapping enriched pathways, including pathways related to inflammation, cell survival, and stress responses (Figure 3C).

### 3.5. qRT-PCR Validation of NanoString-Identified miRNAs in HDM-Treated BEAS-2B Cells and EV-Associated RNA

To independently validate miRNA expression changes identified by NanoString profiling, selected differentially expressed (DE) miRNAs from total cellular RNA and EV-associated RNA were analyzed by qRT-PCR using mature miRNA-specific primers (Table S1).

In total RNA isolated from HDM-treated BEAS-2B cells, qRT-PCR confirmed significant upregulation of miR-1915-3p and miR-126-3p, consistent with the NanoString findings (Figure 4A). Additional miRNAs, including miR-19a-3p and miR-503-5p, showed changes in the same direction but did not reach statistical significance, while miR-191-5p and miR-181a-5p were not significantly altered (Figure 4A).

For EV-associated miRNAs, qRT-PCR confirmed significant upregulation of miR-612, miR-302d-3p, miR-15a-5p, and miR-125b-5p in HDM-treated samples, aligning with the NanoString results (Figure 4B). However, miR-1246 did not show a significant change by qRT-PCR despite being identified as upregulated by NanoString profiling (Figure 4B).

Overall, qRT-PCR validation supported several HDM-responsive miRNAs identified by NanoString profiling, particularly among the EV-associated candidates, while some discrepancies were observed for specific miRNAs such as miR-494-3p, miR-21-5p, and miR-1246.

### 3.6. Assessment of HDM-Responsive EV-Associated miRNAs in BEAS-2B Total Cellular RNA

To determine whether HDM-responsive EV-associated miRNAs exhibited corresponding changes in the cellular compartment, miR-612, miR-302d-3p, miR-1246, miR-15a-5p, and miR-125b-5p were assessed by qRT-PCR in total cellular RNA. None of these miRNAs showed a significant change following HDM treatment (Figure 5). In parallel, several miRNAs identified as differentially expressed in total RNA by NanoString profiling also did not reach statistical significance when examined by qRT-PCR (Figures S6 and S7).

### 3.7. Targeted Analysis of IL-4 Responses in BEAS-2B Cells and EV-Associated RNA

As a secondary targeted analysis, we examined whether selected miRNAs identified from the HDM discovery dataset exhibited similar responses following IL-4 stimulation. Five HDM-responsive EV-associated miRNAs (miR-612, miR-302d-3p, miR-1246, miR-15a-5p, and miR-125b-5p) were analyzed by qRT-PCR in total cellular RNA and EV-associated RNA from IL-4-treated BEAS-2B cells (Figure S7).

In EV-associated RNA, IL-4 treatment resulted in significant decreases in miR-302d-3p and miR-15a-5p, whereas miR-612, miR-1246, and miR-125b-5p exhibited downward trends that did not reach statistical significance (Figure 6A; Figure S7).

A similar pattern was observed in total cellular RNA. miR-612, miR-302d-3p, miR-1246, and miR-15a-5p were significantly decreased following IL-4 treatment, whereas miR-125b-5p showed a nonsignificant reduction (Figure 6B; Figure S7).

Additional miRNAs examined by qRT-PCR also exhibited decreased expression following IL-4 treatment, including miR-19a-3p, miR-19b-3p, miR-126-3p, miR-146a-5p, miR-148b-3p, miR-181a-5p, miR-503-5p, miR-1234-3p, and miR-4454 (Figure 7; Figure S7). In contrast, miR-99a-5p, miR-191-5p, miR-423-3p, miR-424-5p, miR-1915-3p, and miR-4284 were not significantly altered by IL-4 treatment (Figure S8).

Overall, most miRNAs examined by qRT-PCR exhibited reduced expression following IL-4 treatment in both total cellular RNA and EV-associated RNA compared with control samples.

### 3.8. Target-Gene Assessment in GSE158752

To assess the potential clinical relevance of the miRNAs identified in the BEAS-2B model, we examined expression of experimentally validated miRNA target genes in the independent bronchial epithelial RNA-sequencing dataset GSE158752, which includes bronchial epithelial transcriptomic profiles from 17 healthy controls (HC), 25 patients with mild-to-moderate asthma (M), and 25 patients with severe asthma (S).

Several miRNAs, including miR-25-3p, miR-181a-5p, and miR-34a-5p, were downregulated in HDM-treated BEAS-2B cells relative to controls. Correspondingly, expression of BAX, MCL1, and RUNX2 was increased in the mild-to-moderate and/or severe asthma groups compared with healthy controls. Conversely, miR-19b-3p, miR-106b-5p, miR-590-5p, and miR-1234-3p were upregulated following HDM exposure, whereas their validated target gene, STAT3, was significantly decreased in the severe asthma group compared with healthy controls (Table S2, Figure S9A).

We next examined target genes associated with miRNAs differentially expressed in BEAS-2B cells and BEAS-2B-derived EVs. miR-494-3p and miR-21-5p were identified as downregulated in EV-associated RNA, whereas miR-222-3p was downregulated in total cellular RNA. Expression of several validated target genes, including ARNTL, IRAK1, TLR2, ZEB1, and ZEB2, was increased in the severe asthma group relative to healthy controls. In contrast, miR-612, miR-15a-5p, and miR-125b-5p were upregulated in HDM-treated EVs, whereas their validated target genes (AKT2, HMGA2, SNAI1, WNT3A, and YAP1) were decreased in the severe asthma group (Table S3, Figure S9B).

Several miRNAs were similarly dysregulated in both HDM-treated BEAS-2B cells and BEAS-2B-derived EVs. These included downregulated miRNAs (miR-30a-3p, miR-21-5p, miR-25-3p, and miR-93-5p) and upregulated miRNAs (miR-126-3p, miR-1915-3p, miR-15a-5p, miR-125b-5p, miR-106b-5p, miR-302d-3p, miR-146a-5p, miR-19a-3p, and miR-19b-3p). Expression of MYD88 and TGFBR2 was increased, whereas BCL2, CCND2, and SMAD4 were decreased in the severe asthma group relative to healthy controls (Tables S2 and S3, Figure S9C).

A summary of the experimentally validated miRNA-target relationships identified in the BEAS-2B datasets and their corresponding expression patterns in GSE158752 is provided in Table S4.

## 4. Discussion

Accumulating evidence has established the airway epithelium as a central regulator of allergic inflammation through both intracellular signaling and extracellular communication [4,5,11]. miRNAs are important modulators of epithelial responses to allergens, pollutants, and viral stimuli, influencing barrier integrity, cytokine production, epithelial differentiation, and immune-cell recruitment [3,11,40,41,42,43]. In parallel, extracellular vesicles (EVs) have emerged as key mediators of epithelial communication through the transfer of miRNAs, proteins, lipids, and inflammatory signals [3,11,41,42]. Together, these mechanisms position both cellular and EV-associated miRNAs as important regulators of airway inflammation.

In the present study, global NanoString profiling identified distinct cellular and EV-associated miRNA responses to HDM exposure in the BEAS-2B bronchial epithelial cell model. HDM induced substantial intracellular miRNA expression, whereas a smaller but distinct set of miRNAs was altered in EV-associated RNA. A secondary targeted qRT-PCR analysis further showed that several HDM-responsive miRNAs exhibited different expression patterns following IL-4 stimulation, suggesting context-dependent regulation among the selected miRNAs. These findings are consistent with the ability of HDM to activate multiple epithelial stress, inflammatory, and remodeling pathways, including calcium signaling, protease-activated receptors, oxidative stress responses, and alarmin release [6,7,44,45,46,47].

Several of the HDM-regulated miRNAs identified in this study have established roles in asthma-relevant pathways. For example, miR-126-3p has been linked to allergic airway inflammation and Th2 responses, whereas miR-146a-5p functions as an important regulator of NF-κB signaling [18,22,45,48,49,50,51]. Reduced epithelial miR-146a expression has been reported in asthma, while inflammatory stimulation can induce miR-146a as a compensatory anti-inflammatory response [52,53]. The presence of both induced and repressed miRNAs in our dataset therefore suggests broad remodeling of epithelial regulatory networks rather than a uniform activation or suppression of miRNA expression.

Among the identified miRNAs, miR-15a-5p was particularly noteworthy because NanoString profiling revealed decreased cellular abundance but increased EV-associated abundance following HDM exposure. Reduced miR-15a-5p has also been reported following bronchial HDM allergen provocation in patients with allergic asthma and has been associated with greater airway inflammatory responses [47]. Although the cellular decrease was not confirmed by qRT-PCR, EV-associated enrichment of miR-15a-5p was independently validated. Similar discordant cell–EV expression patterns have been reported in other systems and are consistent with differential partitioning of miRNAs between intracellular and EV compartments [15,16,25,27,48,54]. While these observations do not establish active EV sorting, they suggest that EV-associated miRNA abundance may not simply reflect intracellular expression and support further investigation of miR-15a-5p in the context of epithelial intercellular communication.

A major strength of this study is the direct comparison between cellular and EV-associated miRNA signatures. Whereas HDM altered a large number of intracellular miRNAs, only a limited subset was detected as differentially expressed in EV-associated RNA, indicating that EV-associated miRNA abundance does not simply mirror intracellular miRNA levels. This observation is consistent with previous studies suggesting that EV cargo composition is influenced by selective and potentially regulated mechanisms involving RNA-binding proteins, RNA sequence motifs, and vesicle biogenesis pathways [25,26]. Although the present data do not establish a specific sorting mechanism [28], they indicate that cellular and EV-associated miRNA profiles may differ.

The importance of these findings is reinforced by growing evidence that epithelial-derived EVs contribute to allergic sensitization and airway inflammation. Epithelial EVs have been implicated in Th2 polarization, inflammatory signaling, macrophage activation, and epithelial barrier regulation, and EV-associated miRNA profiles have been linked to asthma severity and lung-function impairment [13,14,15,17,18,19,22,23,24]. Collectively, these observations provide biological context for the EV-associated miRNA changes identified in the present study.

Among the EV-associated miRNAs validated by qRT-PCR, miR-612, miR-302d-3p, miR-15a-5p, and miR-125b-5p represent promising candidates for future investigation. miR-125b-5p has been associated with oxidative stress, epithelial inflammation, macrophage activation, airway remodeling, and apoptosis in asthma–COPD overlap. It has also been reported to suppress goblet-cell differentiation through SPDEF and to exhibit reduced abundance in sputum from children with asthma [55,56]. In contrast, miR-125b-5p was enriched in HDM-associated EV RNA in the present study without a corresponding increase in total cellular RNA, emphasizing that EV-associated abundance should not be assumed to reflect intracellular expression patterns directly. These observations identify several EV-associated miRNAs as candidates for future mechanistic studies of allergen-induced intercellular communication.

Targeted analysis of IL-4-treated samples demonstrated a predominantly decreased expression pattern among the miRNAs examined in both cellular and EV-associated RNA. These responses differed from those observed following HDM exposure, suggesting that allergen-associated and cytokine-driven epithelial signaling may regulate miRNA expression differently. Because the IL-4 analysis was limited to selected candidates, these findings should be viewed as preliminary evidence for stimulus-dependent regulation rather than a comprehensive comparison of HDM- and IL-4-responsive miRNA programs.

Bioinformatic analyses further provided insight into the biological relevance of the identified miRNAs. HDM-responsive miRNAs were associated with pathways related to cell-cycle regulation, p53 signaling, FoxO signaling, autophagy, cellular senescence, and inflammatory responses [57,58,59,60]. These pathways are consistent with processes implicated in epithelial injury, repair, remodeling, and chronic airway dysfunction. However, because pathway enrichments are based on predicted and validated target-gene relationships, they identify biological processes potentially influenced by the observed miRNA changes rather than directly demonstrating pathway activation or inhibition.

To explore possible clinical relevance, we examined experimentally validated target genes in the independent GSE158752 bronchial epithelial transcriptomic dataset [38, 39]. Several targets, including RUNX2, TLR2, ZEB2, MYD88, IRAK1, and TGFBR2, have established roles in epithelial inflammatory signaling and airway remodeling and exhibited expression patterns consistent with relationships predicted from the BEAS-2B findings [53,61,62,63,64,65,66,67,68,69,70,71,72,73,74]. At the same time, some targets displayed expression patterns that differed from previous reports, highlighting the heterogeneity of asthma and the influence of disease severity, inflammatory phenotype, treatment exposure, and cellular composition. Accordingly, these analyses should be interpreted as supportive cross-dataset evidence rather than direct validation of individual miRNA-target interactions.

Several limitations should be acknowledged. First, the study was performed exclusively in the immortalized BEAS-2B epithelial cell line, which does not fully reproduce the complexity of differentiated primary human airway epithelium. Second, NanoString profiling was conducted with a relatively modest sample size (n = 6/group), and the findings should therefore be considered exploratory and hypothesis-generating. Third, the study did not directly evaluate functional transfer of specific EV-associated miRNAs or their downstream biological effects in recipient cells. Fourth, although EV-associated markers were assessed, treatment-induced alterations in EV protein cargo were not examined. Finally, the IL-4 analyses were limited to selected miRNAs and were performed at a single concentration and time point. Future studies incorporating primary airway epithelial models, comprehensive cytokine profiling, functional EV-transfer experiments, EV proteomics, and dose–response or time-course analyses will be required to establish the mechanisms and biological consequences of the miRNA signatures identified here.

## 5. Conclusions

Global miRNA profiling identified distinct cellular and EV-associated miRNA responses to HDM exposure in the BEAS-2B bronchial epithelial cell model. HDM induced extensive remodeling of the cellular miRNA landscape, whereas EV-associated RNA exhibited a smaller but distinct set of differentially expressed miRNAs, highlighting differences between intracellular and EV-associated miRNA compartments. Among the identified candidates, miR-612, miR-302d-3p, miR-15a-5p, and miR-125b-5p were independently supported by qRT-PCR and emerged as prominent EV-associated miRNAs following HDM exposure. Targeted analysis further demonstrated that several HDM-responsive miRNAs exhibited distinct expression patterns following IL-4 stimulation, supporting stimulus-dependent regulation of selected epithelial miRNAs. Collectively, these findings identify EV-associated miRNAs as candidates for further study in allergen-induced epithelial communication and provide a foundation for future mechanistic studies and validation in differentiated primary human airway epithelial cell models.

## Figures and Tables

**Figure 1:**
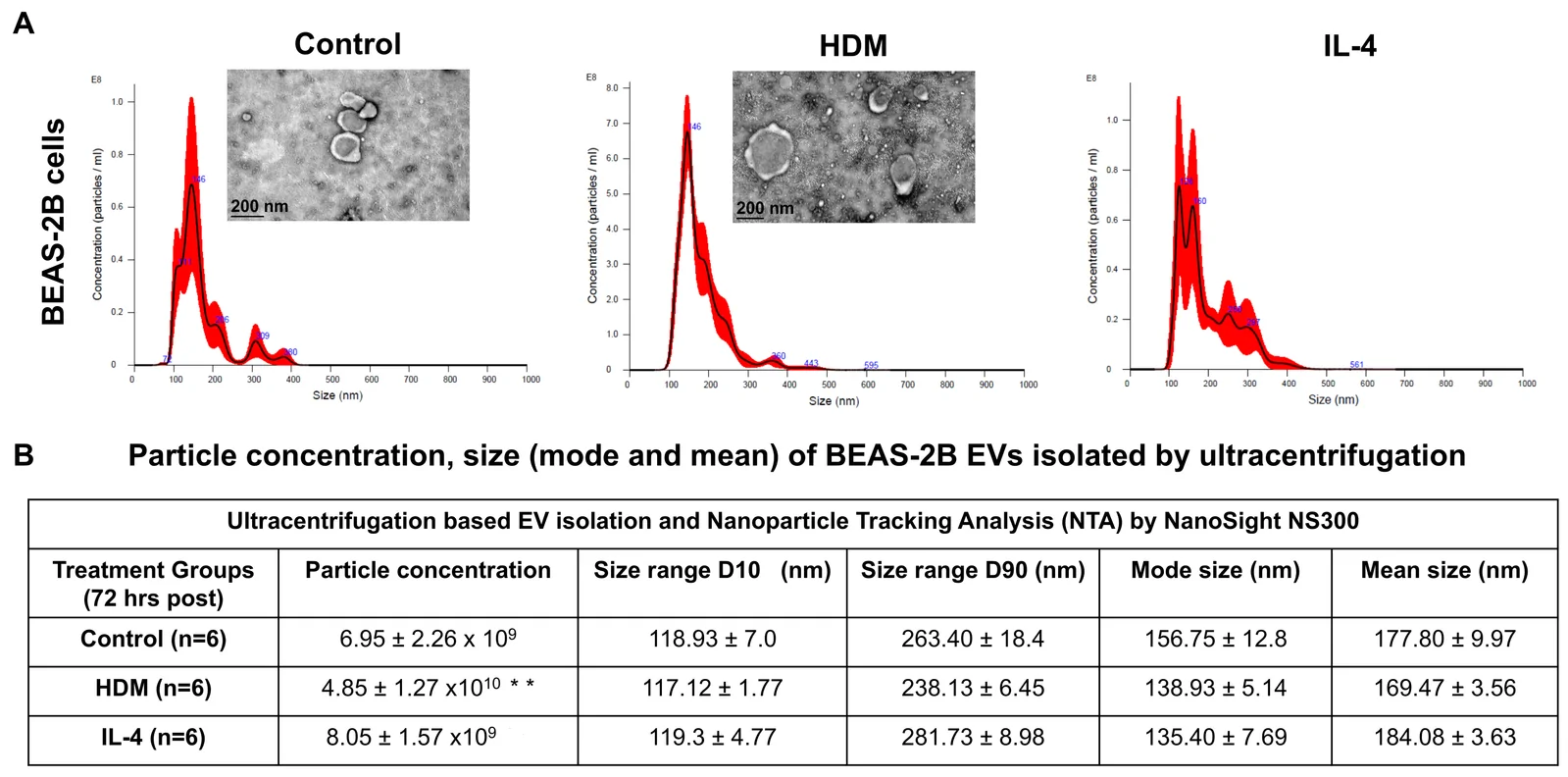
Isolation and characterization of BEAS-2B-derived extracellular vesicles (EVs) from control, house dust mite (HDM) extract-, and IL-4-treated conditioned media. (**A**) Representative nanoparticle tracking analysis (NTA) profiles showing particle concentration and size distribution, along with transmission electron microscopy (TEM) images of EVs isolated from conditioned media of control- and HDM-treated BEAS-2B cells at 72 h post-treatment using differential ultracentrifugation. Scale bar: 200 nm. (**B**) Quantitative summary of NTA data demonstrating treatment-associated differences in particle concentration relative to control, with no significant differences in particle-size distribution. Data are presented as mean ± SEM (n = 6/group) and were analyzed using one-way ANOVA followed by Tukey’s multiple-comparisons test. ** *p* < 0.01, control versus HDM . Statistical annotations indicate comparisons with the corresponding control condition; differences between HDM and IL-4 were not interpreted as comparative measures of stimulus potency.

**Figure 2:**
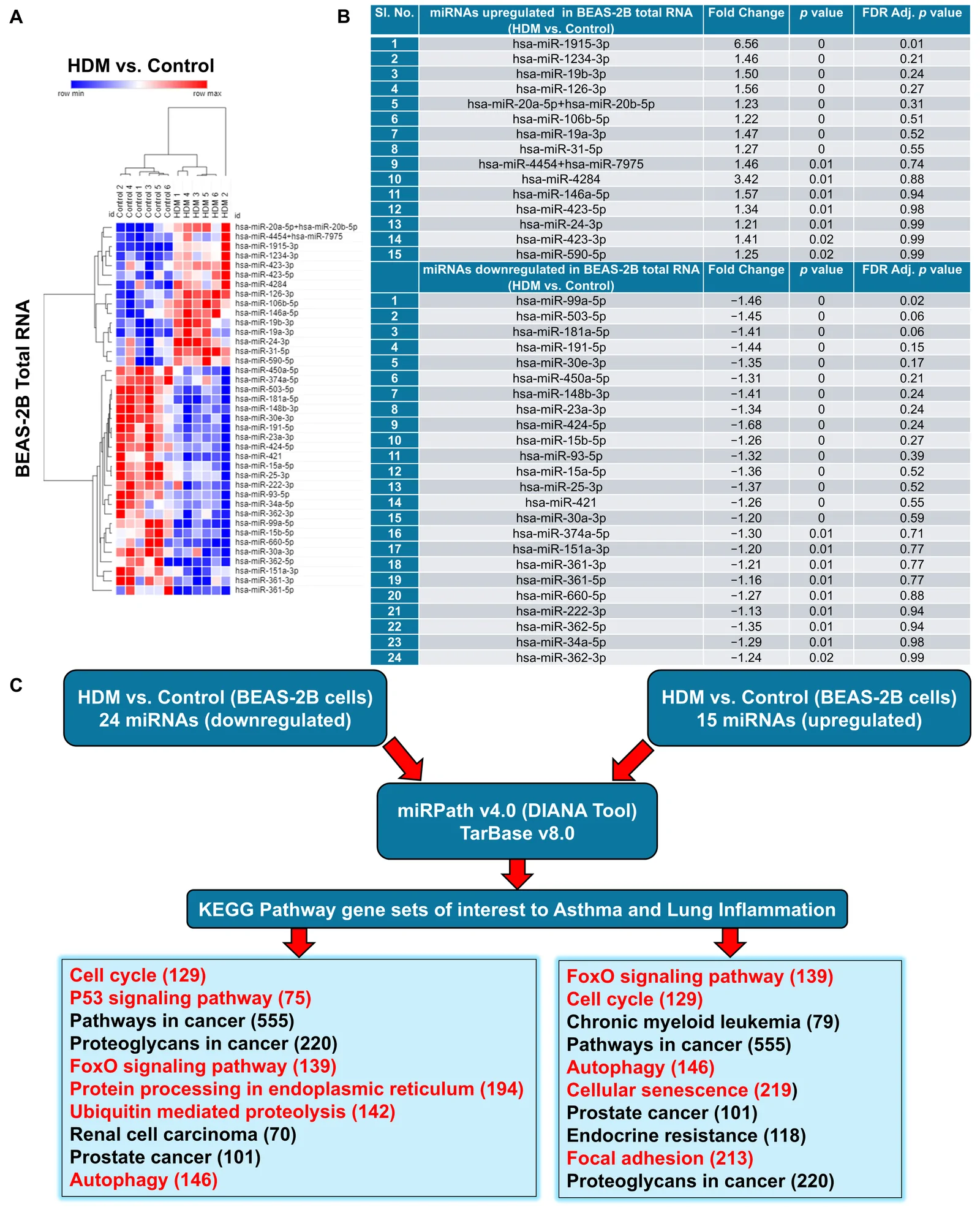
Differentially expressed miRNAs in HDM-treated versus control BEAS-2B cells analyzed by NanoString. (**A**) Heatmap depicting differentially expressed (DE) miRNAs (15 upregulated and 24 downregulated) in BEAS-2B cells treated with HDM for 72 h compared with control cells. Unsupervised hierarchical clustering was performed using the Morpheus tool. (**B**) Pairwise comparison of differentially expressed miRNAs between HDM-treated and control BEAS-2B cells generated using nSolver 4.0 software. Data are presented as fold-change ratios with corresponding nominal *p* values and Benjamini–Hochberg FDR-adjusted *p* values (n = 6/group). Differentially expressed miRNAs met the prespecified abundance criterion (average normalized count ≥80 in at least one comparison group) and a nominal *p* value < 0.05. Fold change and FDR-adjusted *p* values are reported for descriptive and multiple-testing context, respectively. (**C**) KEGG pathway-enrichment analysis performed separately for downregulated and upregulated miRNAs using DIANA miRPath v4.0 with TarBase v8.0. The left panel shows pathways identified from the 24 downregulated cellular miRNAs, whereas the right panel shows pathways identified from the 15 upregulated cellular miRNAs. Numbers in parentheses indicate the number of genes annotated to each corresponding KEGG pathway. KEGG pathways relevant to asthma and inflammation (highlighted in red color text) were enriched based on miRPath analysis of differentially expressed (DE) miRNAs.

**Figure 3:**
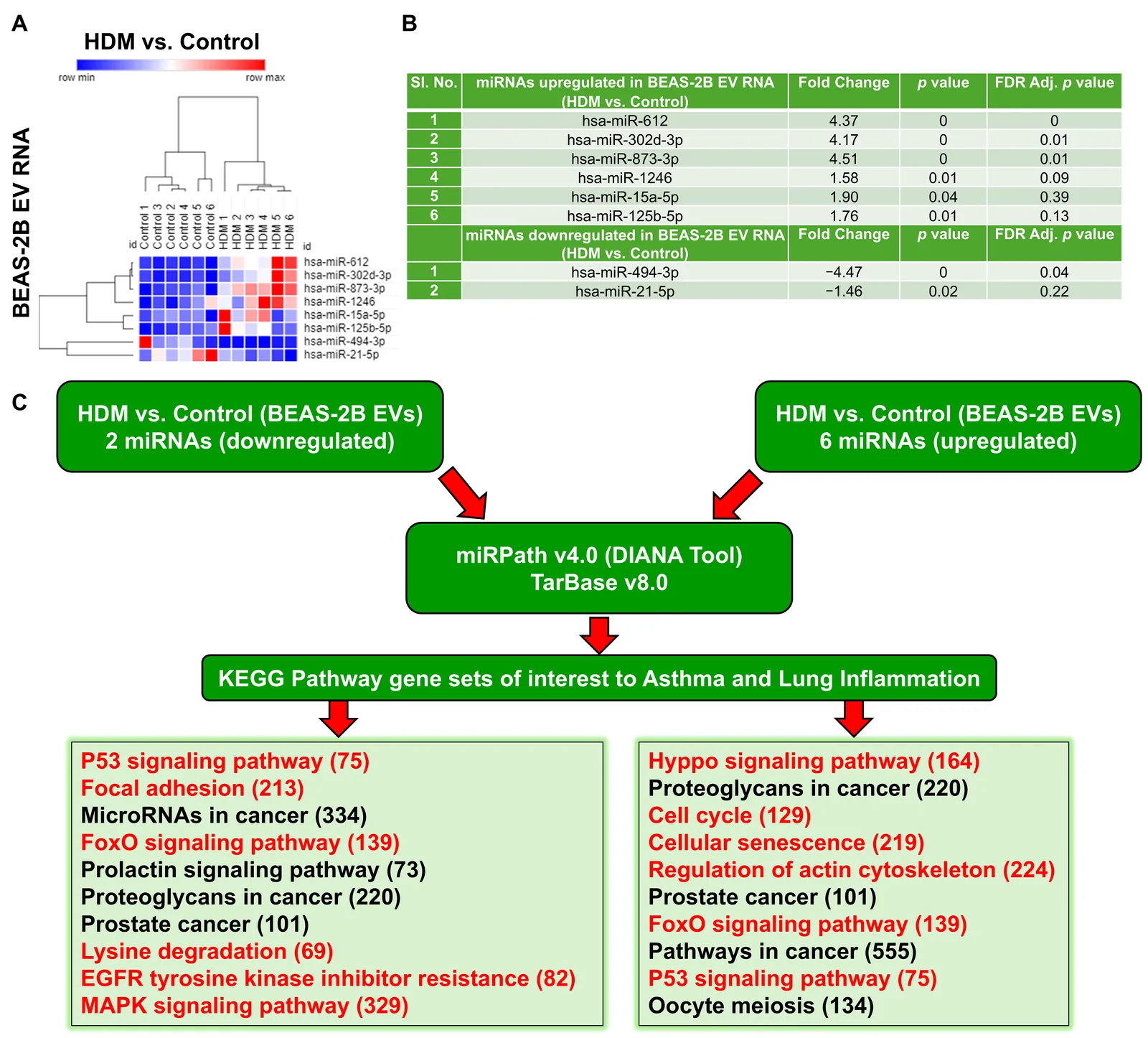
Differentially expressed miRNAs in EV-associated RNA from HDM-treated versus control BEAS-2B-derived EV-enriched preparations analyzed by NanoString. (**A**) Heatmap showing differentially expressed (DE) miRNAs (6 upregulated and 2 downregulated) in EVs isolated from BEAS-2B cells treated with HDM for 72 h compared with control EVs. Unsupervised hierarchical clustering was performed using the Morpheus tool. (**B**) Pairwise comparison of DE miRNAs between HDM-treated and control EV-associated RNA samples generated using nSolver 4.0 software. Data are presented as fold-change ratios with corresponding nominal *p* values and Benjamini–Hochberg FDR-adjusted *p* values (n = 6/group). Differentially expressed miRNAs met the prespecified abundance criterion of an average normalized count ≥80 in at least one comparison group and a nominal *p* value < 0.05. Fold-change values describe the magnitude and direction of the treatment-associated response, whereas FDR-adjusted *p* values are reported to provide multiple-testing context and were not used as the primary criterion for differential-expression classification. (**C**) Bioinformatic pathway analysis of downregulated and upregulated DE miRNAs in HDM-treated versus control BEAS-2B EVs using DIANA miRPath v4.0. KEGG pathway-enrichment analysis performed separately for downregulated and upregulated EV-associated miRNAs using DIANA miRPath v4.0 with TarBase v8.0. The left panel shows pathways identified from the 2 downregulated EV-associated miRNAs, whereas the right panel shows pathways identified from the 6 upregulated EV-associated miRNAs. Numbers in parentheses indicate the number of genes annotated to each corresponding KEGG pathway. KEGG pathways relevant to asthma and inflammation (highlighted in red color text) were enriched based on miRPath analysis of differentially expressed (DE) miRNAs.

**Figure 4:**
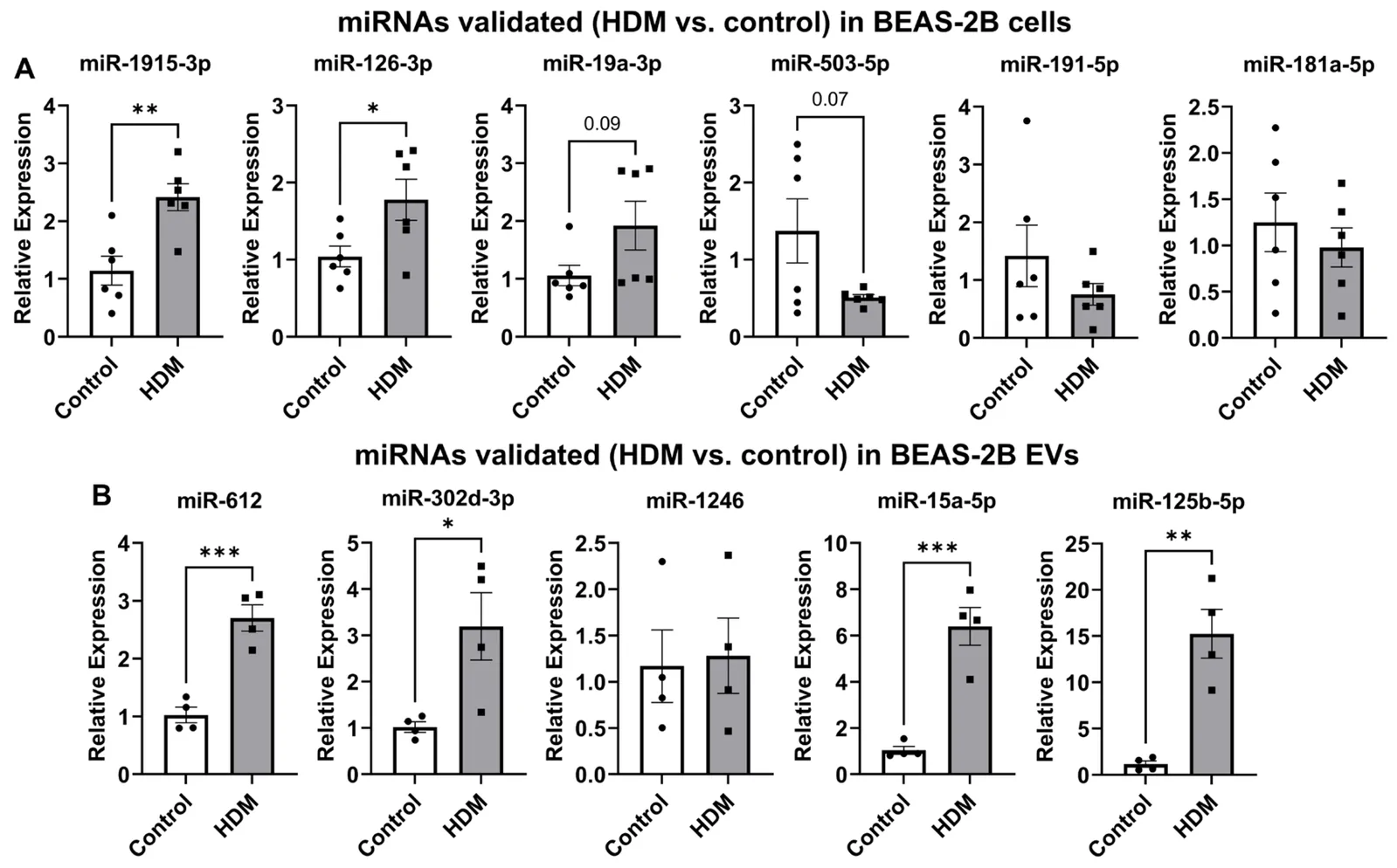
qRT-PCR assessment of selected NanoString-identified miRNAs in HDM-treated BEAS-2B cells and BEAS-2B-derived EV-associated RNA. (**A**) Selected cellular miRNAs identified as differentially expressed by NanoString profiling were independently assessed by quantitative real-time PCR (qRT-PCR) in HDM-treated and control BEAS-2B cells. miR-1915-3p and miR-126-3p showed significant increases by qRT-PCR, whereas the remaining candidates showed nonsignificant trends or no significant change. (**B**) Selected EV-associated miRNAs identified from the NanoString dataset were assessed by qRT-PCR in EV-associated RNA from HDM-treated and control samples. miR-612, miR-302d-3p, miR-15a-5p, and miR-125b-5p showed significant increases, whereas miR-1246 was not significantly changed by qRT-PCR. Data are presented as mean ± SEM (n = 4–6 biological replicates/group). Statistical significance was assessed using an unpaired two-tailed Student’s *t*-test: * *p* < 0.05, ** *p* < 0.01, and *** *p* < 0.001 versus control. Exact *p* values are shown for selected nonsignificant comparisons where indicated.

**Figure 5:**
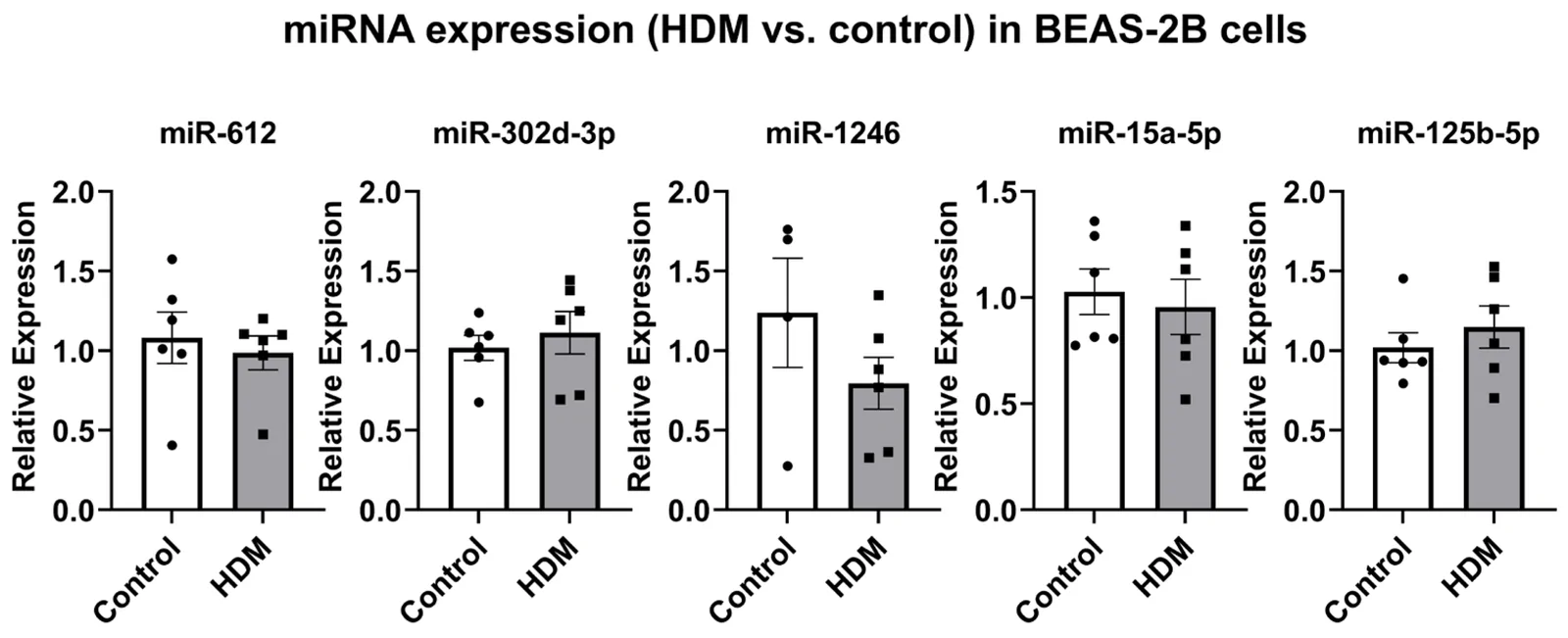
qRT-PCR assessment of selected HDM-responsive EV-associated miRNAs in total cellular RNA from BEAS-2B cells. Selected miRNAs identified as differentially expressed in EV-associated RNA following HDM exposure by NanoString profiling (miR-612, miR-302d-3p, miR-1246, miR-15a-5p, and miR-125b-5p) were independently assessed by quantitative real-time PCR (qRT-PCR) in total cellular RNA from HDM-treated and control BEAS-2B cells. None of the five miRNAs showed a statistically significant change in total cellular RNA following HDM treatment. Data are presented as mean ± SEM (n = 4–6 biological replicates/group), with individual data points shown. Statistical significance was assessed using an unpaired two-tailed Student’s *t*-test.

**Figure 6:**
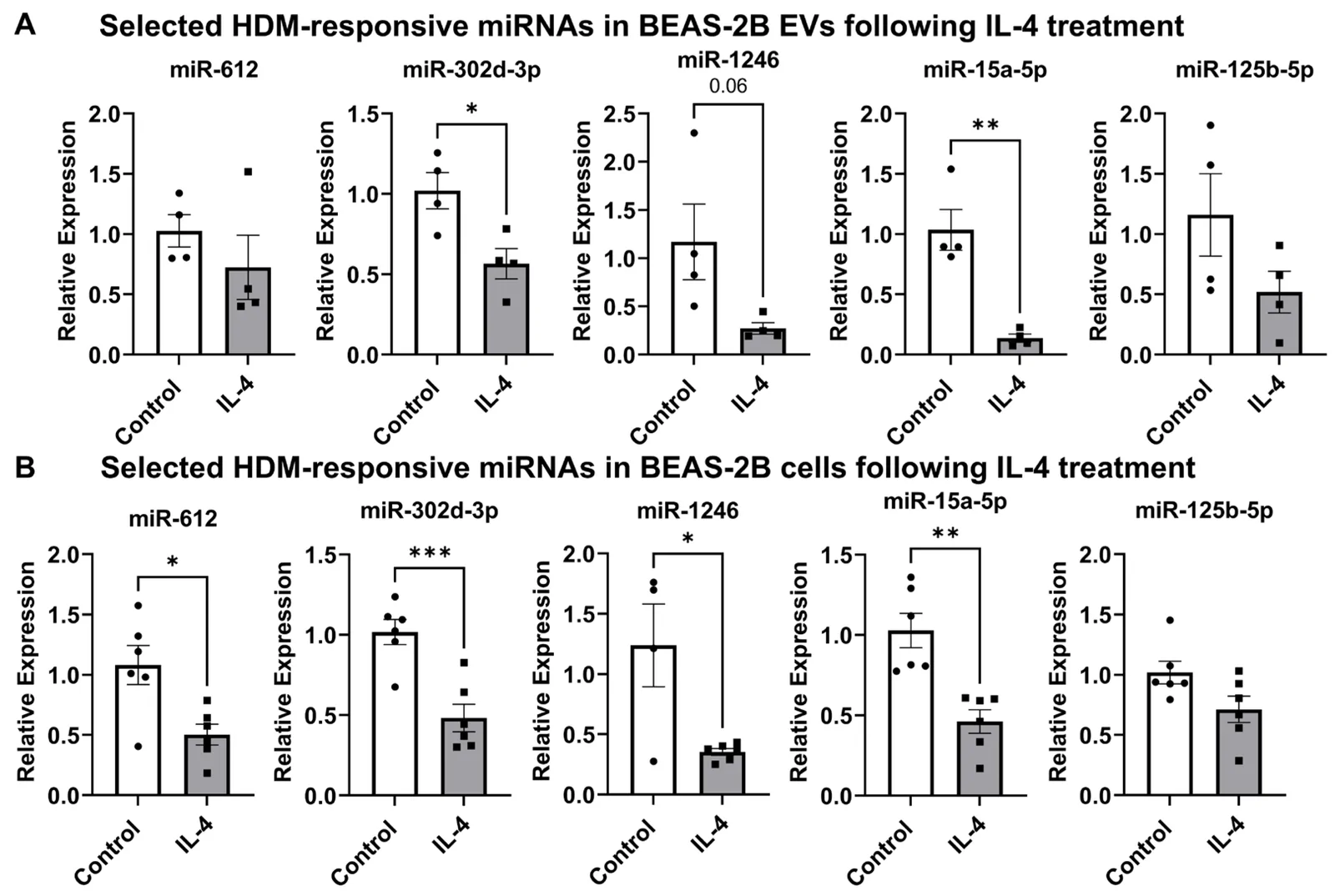
Targeted analysis of HDM-responsive EV-associated miRNAs following IL-4 stimulation. Selected miRNAs identified from the HDM NanoString analysis were evaluated by qRT-PCR following IL-4 treatment. (**A**) Expression of selected miRNAs in EV-associated RNA from IL-4-treated versus control samples. (**B**) Expression of the same miRNAs in total cellular RNA from IL-4-treated versus control BEAS-2B cells. Data are shown as mean ± SEM (n = 4–6 biological replicates/group; * *p* < 0.05, ** *p* < 0.01, *** *p* < 0.001, significant compared to control; unpaired two-tailed Student’s *t*-test was used for pairwise comparison).

**Figure 7:**
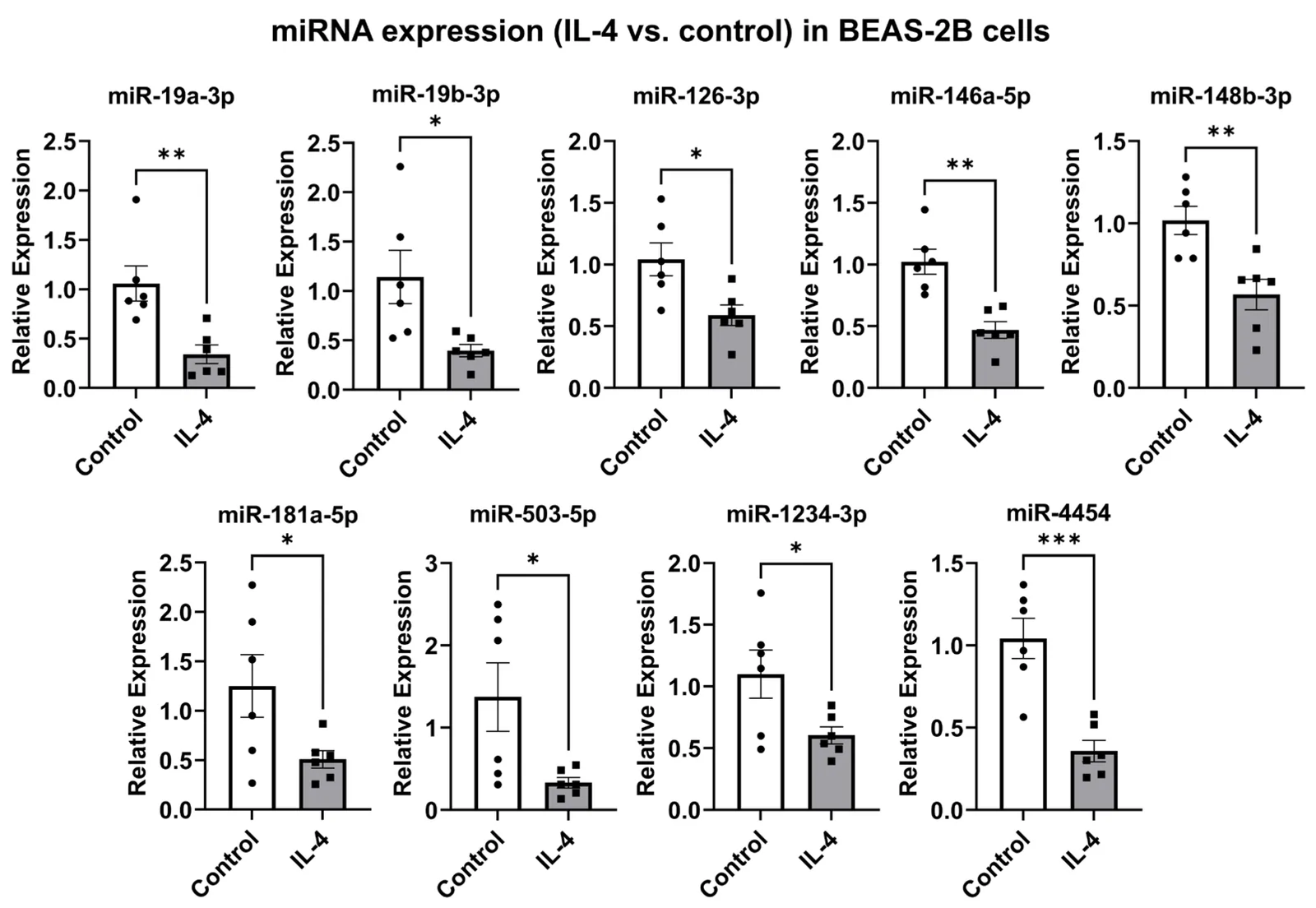
Targeted qRT-PCR analysis of selected miRNAs following IL-4 treatment in BEAS-2B cells. Selected miRNAs were assessed by quantitative real-time PCR (qRT-PCR) in total cellular RNA from IL-4-treated and control BEAS-2B cells. All nine miRNAs examined (miR-19a-3p, miR-19b-3p, miR-126-3p, miR-146a-5p, miR-148b-3p, miR-181a-5p, miR-503-5p, miR-1234-3p, and miR-4454) showed significantly decreased expression following IL-4 treatment compared with control cells. Data are presented as mean ± SEM (n = 6 biological replicates/group), with individual data points shown. Statistical significance was assessed using an unpaired two-tailed *t*-test: * *p* < 0.05, ** *p* < 0.01, *** *p* < 0.001 versus control.

## Data Availability

All data supporting the findings of this study are included in the published article. Additional data is available from the corresponding author upon reasonable request.

## Supplementary Materials

The following supporting information can be downloaded at https://www.mdpi.com/article/10.3390/cells15191744/s1. Figure S1. Protein-based characterization of BEAS-2B-derived EV-enriched preparations Total protein content and EV-associated markers in isolated BEAS-2B-derived EVs were assessed by stain-free gel electrophoresis and immunoblotting. (A) Representative stain-free gel demonstrating the total protein profile of BEAS-2B-derived EVs isolated by ultracentrifugation. (B) Representative immunoblots showing enrichment of EV-associated markers, including the endosomal sorting protein TSG101, cytosolic adaptor protein Syntenin-1, and tetraspanin CD63 (glycosylated form). The absence of the endoplasmic reticulum proteins GRP94 and calnexin indicates minimal contamination from cellular organelles. Figure S2. Uptake of fluorescently labeled BEAS-2B-derived EV-associated material by recipient BEAS-2B cells. Control BEAS-2B-derived EV-enriched preparations isolated from conditioned medium were fluorescently labeled using the ExoGlow™ membrane EV labeling kit and incubated with recipient BEAS-2B cells cultured on 35-mm glass-bottom dishes. Representative confocal microscopy images acquired 24 h after incubation are shown as fluorescence-only images (left) and corresponding differential interference contrast (DIC)/fluorescence merged images (right). Punctate and perinuclear-associated green fluorescence was observed in recipient BEAS-2B cells following incubation with labeled EV preparations. The merged images are included to demonstrate the spatial relationship between cellular morphology and the fluorescent signal. Two representative fields are shown. Figure S3. Differential expression of miRNAs in HDM-treated versus control BEAS-2B cells assessed by NanoString analysis. Box-and-whisker plots showing normalized NanoString counts for differentially expressed (DE) miRNAs in HDM-treated versus control BEAS-2B cells. (A) Fifteen miRNAs significantly upregulated and (B) Twenty-four miRNAs significantly downregulated following HDM treatment compared with control. Normalized miRNA counts are presented to illustrate expression differences between groups. Data are shown as mean ± SEM (n = 6/group). Statistical significance was determined using an unpaired two-tailed *t*-test: * *p* < 0.05, ** *p* < 0.01, *** *p* < 0.001 versus Control. Figure S4. Differential expression of miRNAs in HDM-treated versus control BEAS-2B-derived EVs assessed by NanoString analysis. Box-and-whisker plots showing normalized NanoString counts for differentially expressed (DE) miRNAs in EVs isolated from HDM-treated versus control BEAS-2B cells. (A) Six miRNAs were significantly upregulated and (B) Two miRNAs were significantly downregulated in HDM-treated EVs compared with control EVs. Overall, a limited number of miRNAs were differentially expressed in EVs following HDM treatment. Data are presented as mean ± SEM (n = 6/group). Statistical significance was determined using an unpaired two-tailed *t*-test: * *p* < 0.05, ** *p* < 0.01, *** *p* < 0.001 versus control. Figure S5. Distinct miRNA expression signatures in HDM-treated versus control BEAS-2B cells and BEAS-2B-derived EVs analyzed by NanoString nCounter Human v3 miRNA assay. (A) Venn diagram illustrating differentially expressed (DE) miRNAs that are significantly upregulated or downregulated in BEAS-2B cells and/or EVs following HDM treatment compared with control. The diagram summarizes DE miRNAs identified by nSolver 4.0 software analysis, as presented in Figure 2 and Figure 3. Upregulated miRNAs are indicated in red, and downregulated miRNAs are indicated in blue. miR‑15a‑5p, which exhibits differential expression in both BEAS-2B cells and EVs with opposite directionality, is underlined. (B) Principal component analysis (PCA) of normalized miRNA expression profiles from HDM-treated and control BEAS-2B cells and EVs. PCA was performed using normalized NanoString miRNA counts generated by nSolver analysis. Samples cluster according to treatment group, with stronger intra‑group clustering observed in BEAS-2B cells compared with EV samples. Figure S6. qRT-PCR assessment of additional NanoString-identified HDM-responsive miRNAs in BEAS-2B cells.Selected miRNAs identified as differentially expressed by NanoString profiling were independently assessed by qRT-PCR in total cellular RNA from HDM-treated and control BEAS-2B cells. None of the miRNAs shown reached statistical significance by qRT-PCR.Data are presented as mean ± SEM with individual biological replicates shown (n = 6/group). Statistical significance was assessed using an unpaired two-tailed Student’s *t*-test. Figure S7. Integrated summary of HDM- and IL-4-associated miRNA responses assessed in BEAS-2B cells and EV-associated RNA.The left panels summarize selected HDM-responsive miRNAs identified by NanoString profiling and their subsequent qRT-PCR assessment in cellular and EV-associated RNA. Arrows indicate statistically significant directional changes (red ↑, increased; blue ↓, decreased), whereas NS denotes no statistically significant change. The right panels summarize targeted qRT-PCR responses of selected HDM-responsive miRNAs following IL-4 stimulation in BEAS-2B cellular and EV-associated RNA; IL-4 samples were not subjected to global NanoString profiling. The schematic is intended to distinguish NanoString discovery findings from independent qRT-PCR support and targeted IL-4 follow-up rather than to imply comprehensive profiling of the IL-4 miRNA landscape. Figure S8. qRT-PCR assessment of selected miRNAs not significantly altered following IL-4 treatment in BEAS-2B cells. Selected HDM-responsive miRNAs were assessed by qRT-PCR in total cellular RNA from IL-4-treated and control BEAS-2B cells. None of the miRNAs shown reached statistical significance following IL-4 treatment. Data are presented as mean ± SEM with individual biological replicates shown (n = 6/group). Statistical significance was assessed using an unpaired two-tailed Student’s *t*-test. Figure S9. Validation of predicted mRNA target genes associated with differentially expressed miRNAs in BEAS-2B cells and EVs following HDM exposure using the publicly available RNA-seq dataset GSE158752. (A) Expression of selected mRNA targets predicted from differentially expressed miRNAs identified in HDM-treated versus control BEAS-2B cells. (B) Expression of selected mRNA targets predicted from differentially expressed miRNAs identified in HDM-treated versus control BEAS-2B-derived EVs. (C) Expression of selected mRNA targets predicted from differentially expressed miRNAs identified in both HDM-treated BEAS-2B cells and BEAS-2B-derived EVs compared with their respective controls. Target genes were selected based on overlaps with predicted miRNA targets from BEAS-2B cells and/or BEAS-2B-derived EVs and on reported regulatory relationships supported by the literature. Data are presented as box-and-whisker plots (minimum to maximum) with individual data points representing normalized RNA-seq counts for each sample. Statistical significance was assessed by one-way ANOVA followed by Tukey’s multiple-comparison test. * *p* < 0.05, ** *p* < 0.01, *** *p* < 0.001 versus healthy controls (HC). Table S1. List of miRNA primers used in this study. Table S2. Experimentally validated target genes of differentially expressed miRNAs identified in HDM-treated versus control BEAS-2B cells based on miRTarBase v9.0. Table S3. Experimentally validated target genes of differentially expressed miRNAs identified in HDM-treated versus control BEAS-2B-derived EVs based on miRTarBase v9.0. Table S4. Asthma relevance of Figure S9 target genes differentially expressed in severe asthma compared with healthy controls.
