# Supplementary material for: House Dust Mite Induces Distinct Cellular and Extracellular Vesicle-Associated miRNA Signatures in BEAS-2B Cells: Targeted Analysis of IL-4 Responses
Source: Cells. 2026 Sep 24;15(19):1744. doi: 10.3390/cells15191744 (PMC13664636; doi:10.3390/cells15191744)
Supplement: Supplementary file 1 [file cells-15-01744-s001.zip › EV MS Supplemental Figures S1-S9_R1.pdf]

Supplemental Figures S1-S9

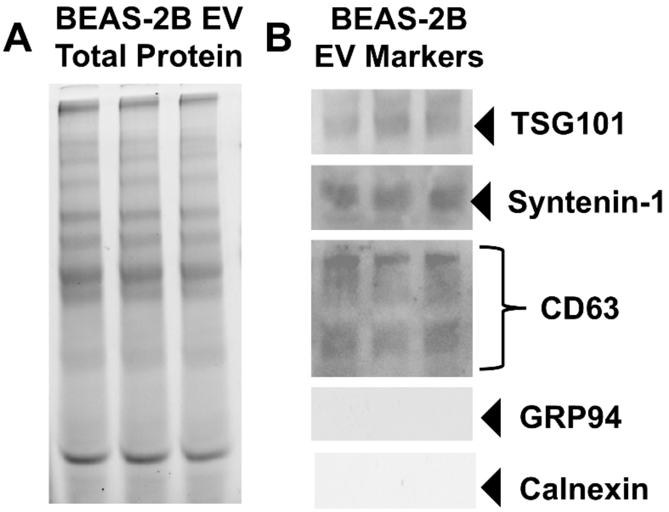

Figure S1. Protein-based characterization of BEAS-2B-derived EV-enriched preparations Total protein content and EV-associated markers in isolated BEAS-2B-derived EVs were assessed by stain-free gel electrophoresis and immunoblotting. (A) Representative stain-free gel demonstrating the total protein profile of BEAS-2B-derived EVs isolated by ultracentrifugation. (B) Representative immunoblots showing enrichment of EV-associated markers, including the endosomal sorting protein TSG101, cytosolic adaptor protein Syntenin-1, and tetraspanin CD63 (glycosylated form). The absence of the endoplasmic reticulum proteins GRP94 and calnexin indicates minimal contamination from cellular organelles.

Supplemental Figures S1-S9

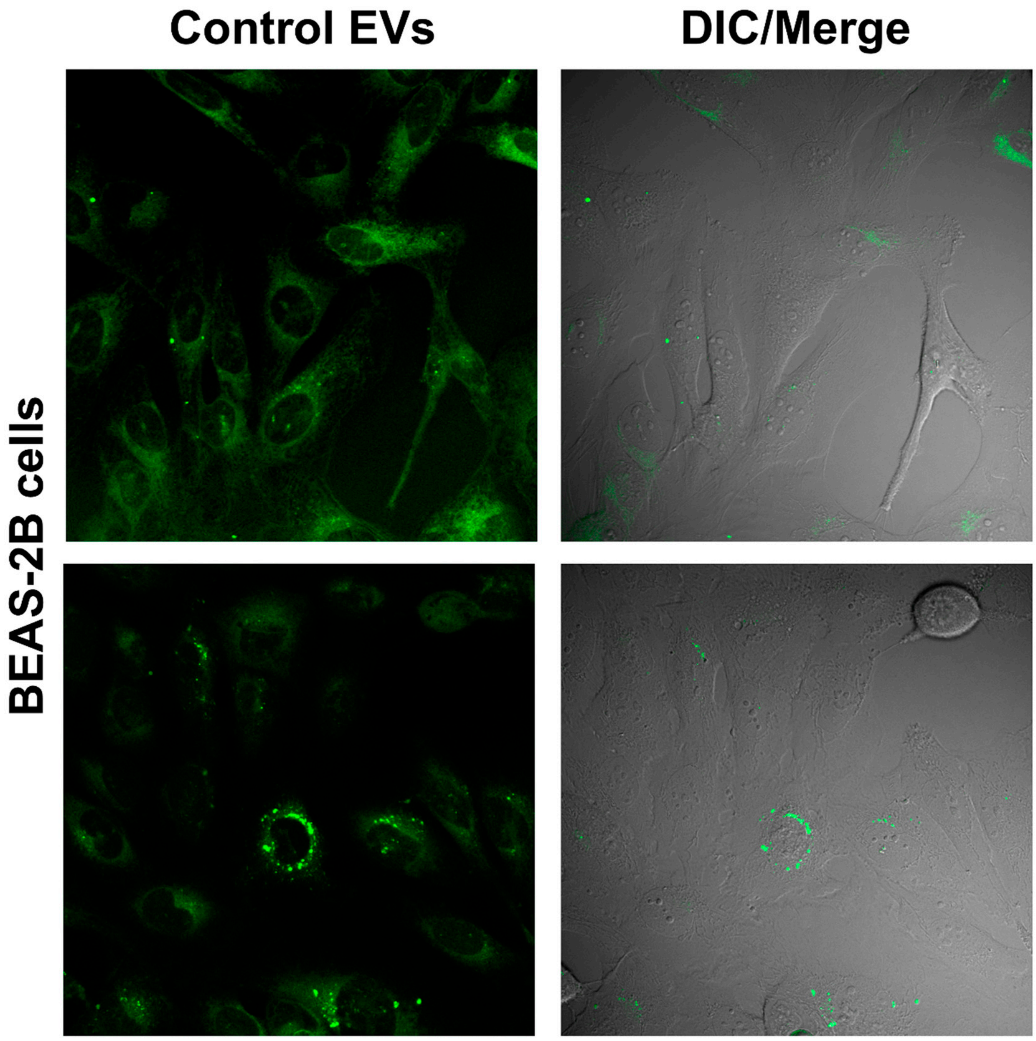

Figure S2. Uptake of fluorescently labeled BEAS-2B-derived EV-associated material by recipient BEAS-2B cells. Control BEAS-2B-derived EV-enriched preparations isolated from conditioned medium were fluorescently labeled using the ExoGlow™ membrane EV labeling kit and incubated with recipient BEAS-2B cells cultured on 35-mm glass-bottom dishes. Representative confocal microscopy images acquired 24 h after incubation are shown as fluorescence-only images (left) and corresponding differential interference contrast (DIC)/fluorescence merged images (right). Punctate and perinuclear-associated green fluorescence was observed in recipient BEAS-2B cells following incubation with labeled EV preparations. The merged images are included to demonstrate the spatial relationship between cellular morphology and the fluorescent signal. Two representative fields are shown.

Supplemental Figures S1-S9

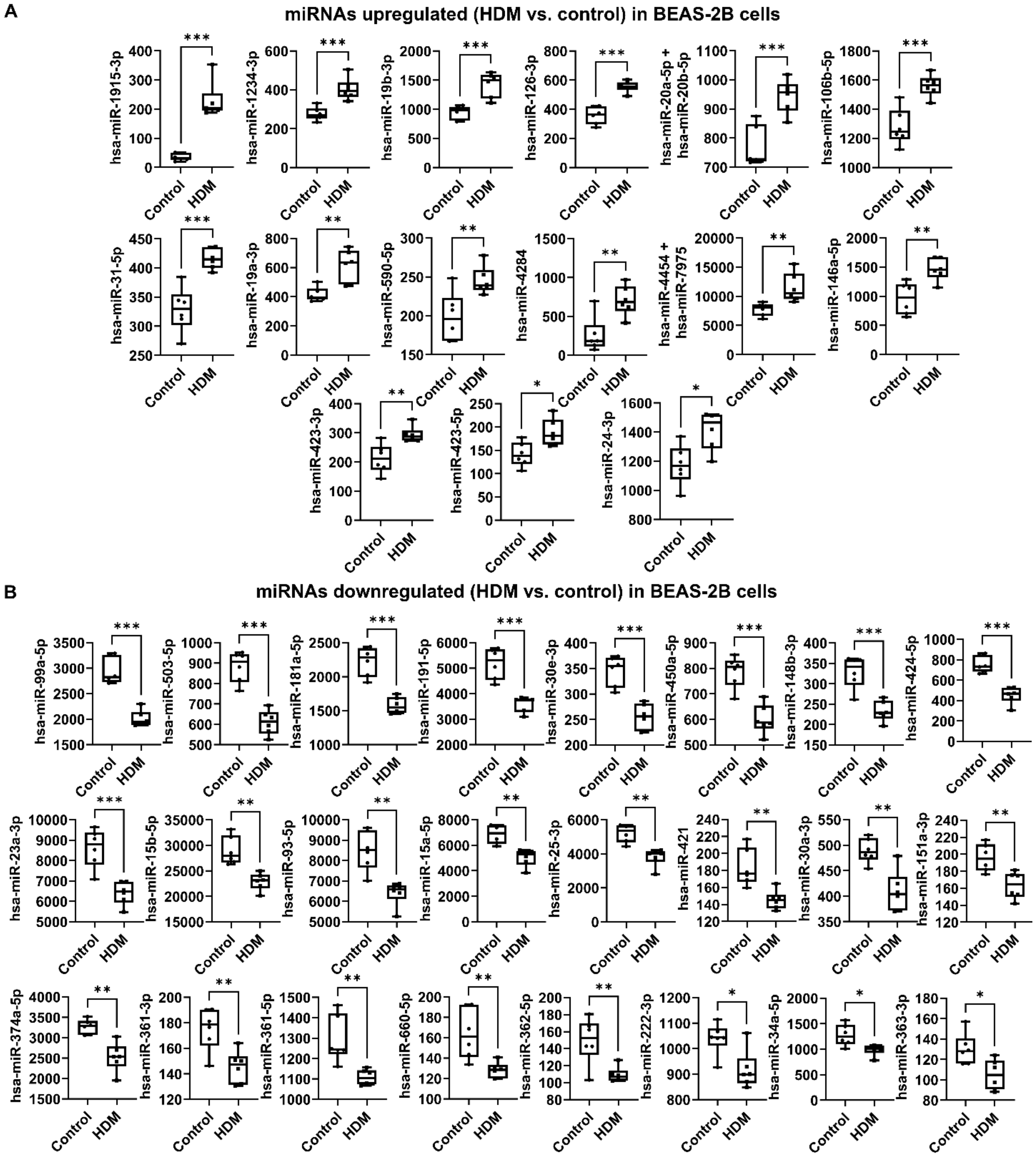

Figure S3. Differential expression of miRNAs in HDM-treated versus control BEAS-2B cells assessed by NanoString analysis. Box-and-whisker plots showing normalized NanoString counts for differentially expressed (DE) miRNAs in HDM-treated versus control BEAS-2B cells. (A) Fifteen miRNAs significantly upregulated and (B) Twenty-four miRNAs significantly downregulated following HDM treatment compared with control. Normalized miRNA counts are presented to illustrate expression differences between groups. Data are shown as mean ± SEM (n = 6/group). Statistical significance was determined using an unpaired two-tailed *t*-test: * $p < 0.05$, ** $p < 0.01$, *** $p < 0.001$ versus Control.

Supplemental Figures S1-S9

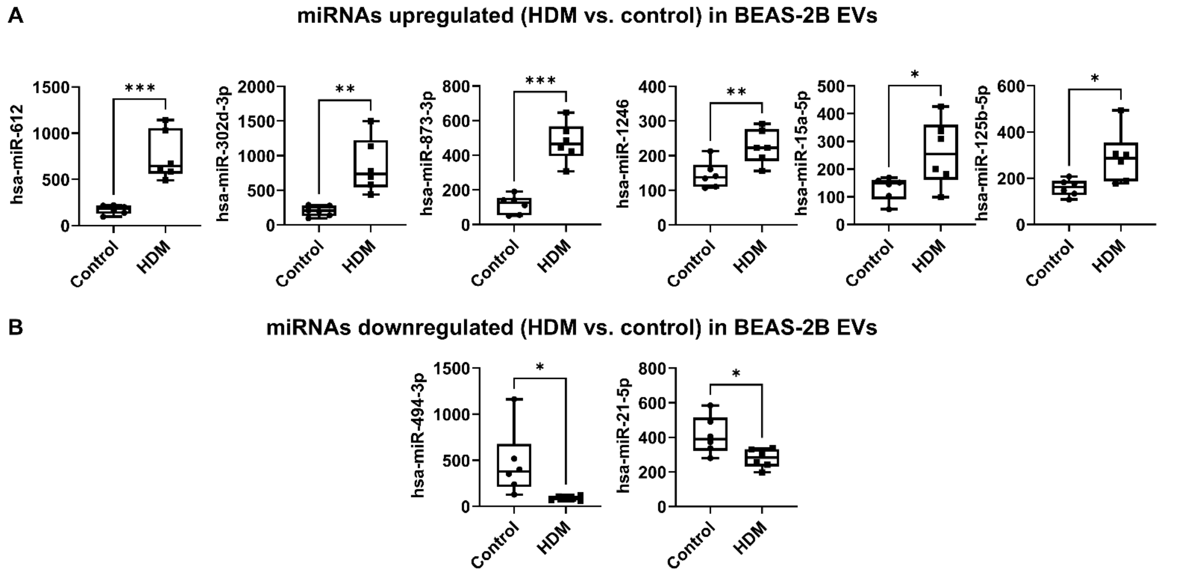

Figure S4. Differential expression of miRNAs in HDM-treated versus control BEAS-2B-derived EVs assessed by NanoString analysis. Box-and-whisker plots showing normalized NanoString counts for differentially expressed (DE) miRNAs in EVs isolated from HDM-treated versus control BEAS-2B cells. (A) Six miRNAs were significantly upregulated and (B) Two miRNAs were significantly downregulated in HDM-treated EVs compared with control EVs. Overall, a limited number of miRNAs were differentially expressed in EVs following HDM treatment. Data are presented as mean ± SEM (n = 6/group). Statistical significance was determined using an unpaired two-tailed *t*-test: * $p < 0.05$, ** $p < 0.01$, *** $p < 0.001$ versus control.

Supplemental Figures S1-S9

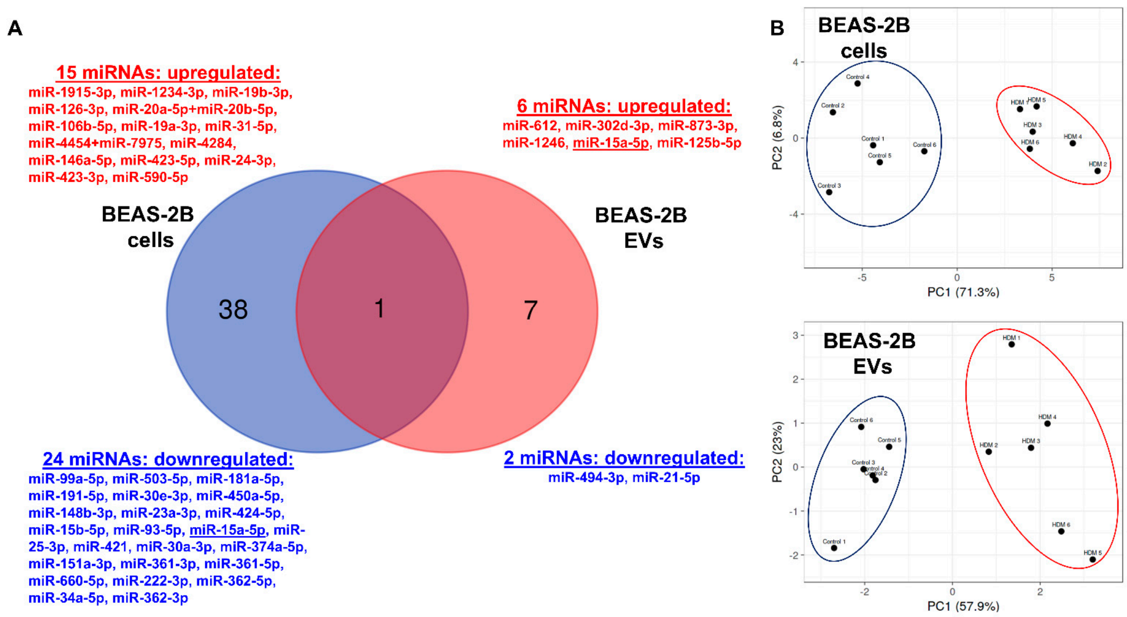

Figure S5. Distinct miRNA expression signatures in HDM-treated versus control BEAS-2B cells and BEAS-2B-derived EVs analyzed by NanoString nCounter Human v3 miRNA assay. (A) Venn diagram illustrating differentially expressed (DE) miRNAs that are significantly upregulated or downregulated in BEAS-2B cells and/or EVs following HDM treatment compared with control. The diagram summarizes DE miRNAs identified by nSolver 4.0 software analysis, as presented in **Figures 2 and 3**. Upregulated miRNAs are indicated in red, and downregulated miRNAs are indicated in blue. miR-15a-5p, which exhibits differential expression in both BEAS-2B cells and EVs with opposite directionality, is underlined. (B) Principal component analysis (PCA) of normalized miRNA expression profiles from HDM-treated and control BEAS-2B cells and EVs. PCA was performed using normalized NanoString miRNA counts generated by nSolver analysis. Samples cluster according to treatment group, with stronger intra-group clustering observed in BEAS-2B cells compared with EV samples.

Supplemental Figures S1-S9

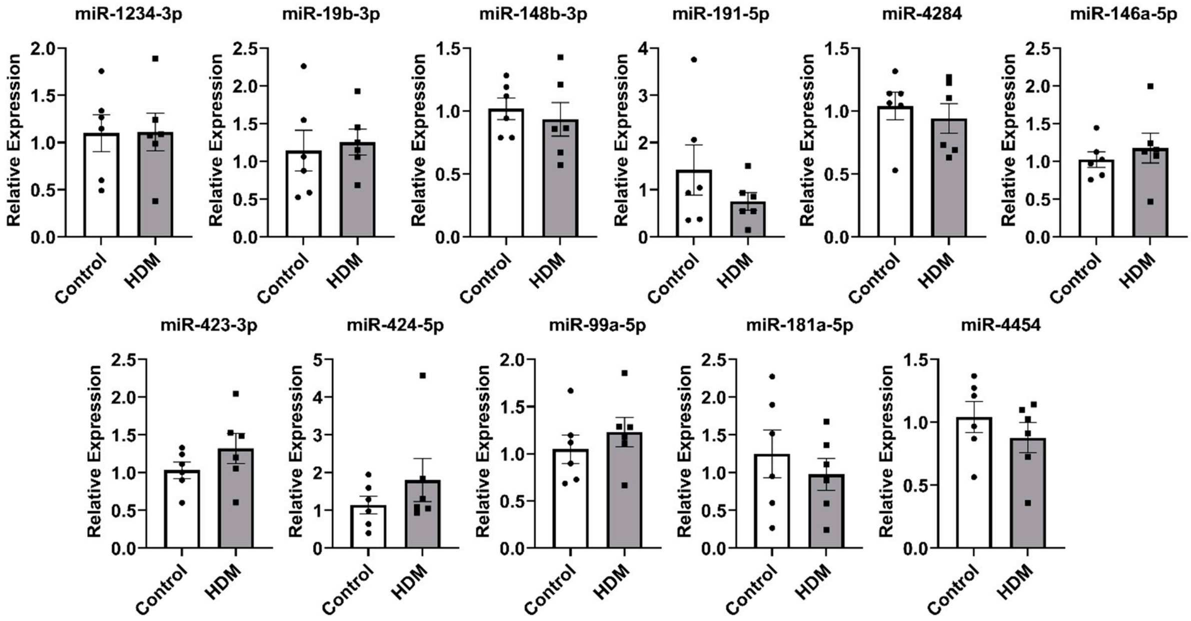

Figure S6. qRT-PCR assessment of additional NanoString-identified HDM-responsive miRNAs in BEAS-2B cells. Selected miRNAs identified as differentially expressed by NanoString profiling were independently assessed by qRT-PCR in total cellular RNA from HDM-treated and control BEAS-2B cells. None of the miRNAs shown reached statistical significance by qRT-PCR. Data are presented as mean ± SEM with individual biological replicates shown (n = 6/group). Statistical significance was assessed using an unpaired two-tailed Student's *t*-test.

Supplemental Figures S1-S9

DE miRNAs in BEAS-2B cells

(HDM vs. Control)

miRNAs	NanoString	qRT-PCR
miR-1915-3p	↑	↑
miR-126-3p	↑	↑
miR-19a-3p	↑	NS
miR-503-5p	↓	NS
miR-191-5p	↓	NS
miR-181a-5p	↓	NS

DE miRNAs in BEAS-2B cells

(IL-4 vs. Control)

miRNAs	qRT-PCR
miR-19a-3p	↓
miR-19b-3p	↓
miR-126-3p	↓
miR-146a-5p	↓
miR-148b-3p	↓
miR-181a-5p	↓
miR-503-5p	↓
miR-1234-3p	↓
miR-4454	↓

DE miRNAs in BEAS-2B EVs

(HDM vs. Control)

miRNAs	NanoString	qRT-PCR
miR-612	↑	↑
miR-302d-3p	↑	↑
miR-1246	↑	NS
miR-15a-5p	↑	↑
miR-125b-5p	↑	↑
miR-494-3p	↓	NS
miR-21-5p	↓	NS

DE miRNAs in BEAS-2B EVs

(IL-4 vs. Control)

miRNAs	qRT-PCR
miR-612	NS
miR-302d-3p	↓
miR-1246	NS
miR-15a-5p	↓
miR-125b-5p	NS

Figure S7. Integrated summary of HDM- and IL-4-associated miRNA responses assessed in BEAS-2B cells and EV-associated RNA. The left panels summarize selected HDM-responsive miRNAs identified by NanoString profiling and their subsequent qRT-PCR assessment in cellular and EV-associated RNA. Arrows indicate statistically significant directional changes (red ↑, increased; blue ↓, decreased), whereas NS denotes no statistically significant change. The right panels summarize targeted qRT-PCR responses of selected HDM-responsive miRNAs following IL-4 stimulation in BEAS-2B cellular and EV-associated RNA; IL-4 samples were not subjected to global NanoString profiling. The schematic is intended to distinguish NanoString discovery findings from independent qRT-PCR support and targeted IL-4 follow-up rather than to imply comprehensive profiling of the IL-4 miRNA landscape.

Supplemental Figures S1-S9

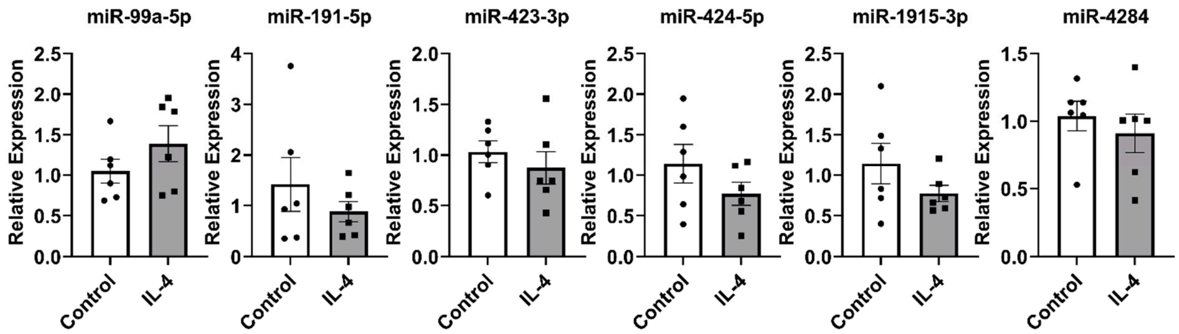

Figure S8. qRT-PCR assessment of selected miRNAs not significantly altered following IL-4 treatment in BEAS-2B cells. Selected HDM-responsive miRNAs were assessed by qRT-PCR in total cellular RNA from IL-4-treated and control BEAS-2B cells. None of the miRNAs shown reached statistical significance following IL-4 treatment. Data are presented as mean ± SEM with individual biological replicates shown (n = 6/group). Statistical significance was assessed using an unpaired two-tailed Student's *t*-test.

Supplemental Figures S1-S9

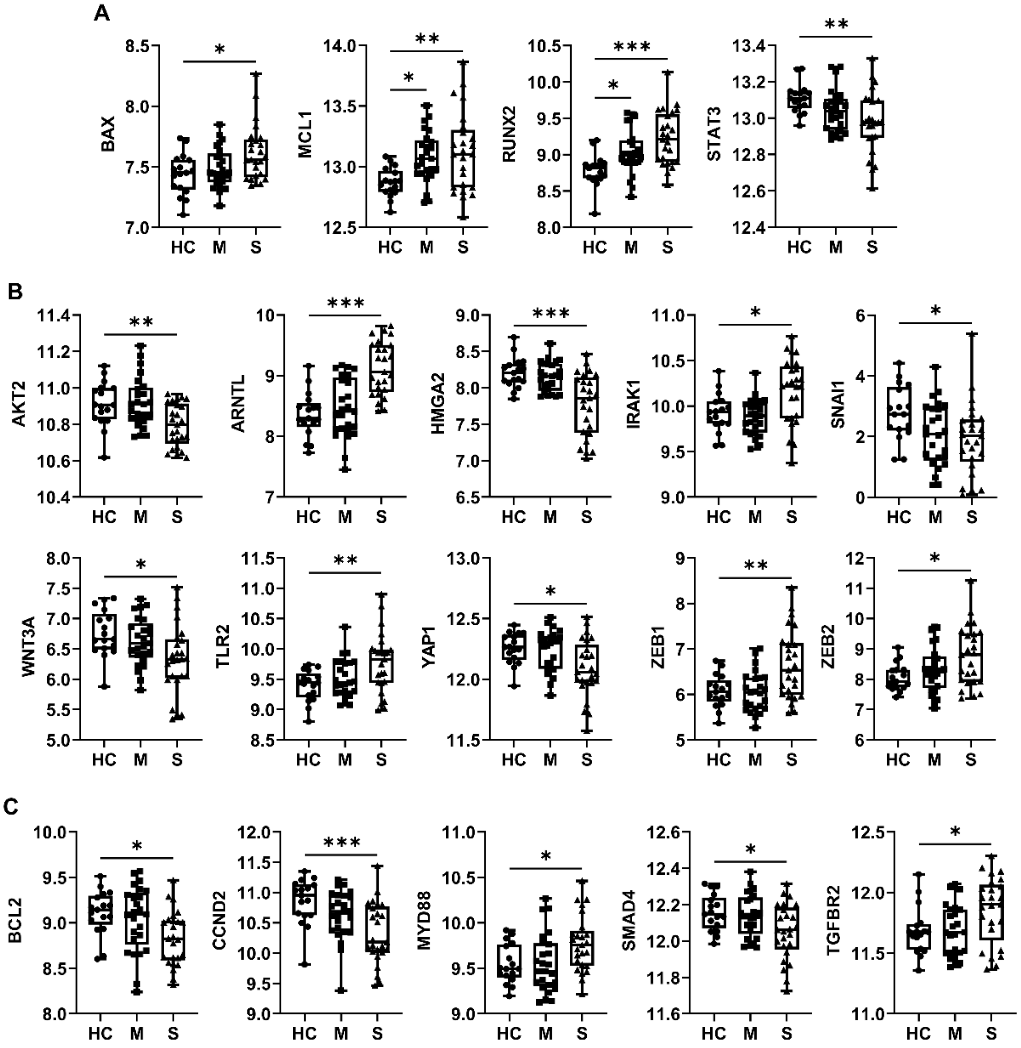

Figure S9. Validation of predicted mRNA target genes associated with differentially expressed miRNAs in BEAS-2B cells and EVs following HDM exposure using the publicly available RNA-seq dataset GSE158752. (A) Expression of selected mRNA targets predicted from differentially expressed miRNAs identified in HDM-treated versus control BEAS-2B cells. (B) Expression of selected mRNA targets predicted from differentially expressed miRNAs identified in HDM-treated versus control BEAS-2B-derived EVs. (C) Expression of selected mRNA targets predicted from differentially expressed miRNAs identified in both HDM-treated BEAS-2B cells and BEAS-2B-derived EVs compared with their respective controls. Target genes were selected based on overlaps with predicted miRNA targets from BEAS-2B cells and/or BEAS-2B-derived EVs and on reported regulatory relationships supported by the literature. Data are presented as box-and-whisker plots (minimum to maximum) with individual data points representing normalized RNA-seq counts for each sample. Statistical significance was assessed by one-way ANOVA followed by Tukey's multiple-comparison test. * $p < 0.05$, ** $p < 0.01$, *** $p < 0.001$ versus healthy controls (HC).
