# Supplementary material for: House Dust Mite Induces Distinct Cellular and Extracellular Vesicle-Associated miRNA Signatures in BEAS-2B Cells: Targeted Analysis of IL-4 Responses
Source: Cells. 2026 Sep 24;15(19):1744. doi: 10.3390/cells15191744 (PMC13664636; doi:10.3390/cells15191744)
Supplement: Supplementary file 1 [file cells-15-01744-s001.zip › EV MS Supplemental Tables S1-S4_R1.pdf]

Table S1. List of miRNA primers used in this study.

Target /miRNA	Sequence
hsa-miR-1915-3P	5’-CCCCAGGGCGACGCGGCGGG-3’
hsa-miR-126-3p	5’-TCGTACCGTGAGTAATAATGCG-3’
hsa-miR-19a-3p	5’-TGTGCAAATCTATGCAAAACTGA-3’
hsa-miR-503-5p	5’-TAGCAGCGGGAACAGTTCTGCAG-3’
hsa-miR-191-5p	5’-CAACGGAATCCCAAAAGCAGCTG-3’
hsa-miR-181a-5p	5’-AACATTCAACGCTGTCGGTGAGT-3’
hsa-miR-612	5’-GCTGGGCAGGGCTTCTGAGCTCCTT-3’
hsa-miR-302d-3p	5’-TAAGTGCTTCCATGTTTGAGTGT-3’
hsa-miR-873-3p	5’-GGAGACTGATGAGTCCCGGGA-3’
hsa-miR-1246	5’-AATGGATTTTTGGAGCAGG-3’
hsa-miR-15a-5p	5’-TAGCAGCACATAATGGTTTGTG-3’
hsa-miR-125b-5p	5’-TCCCTGAGACCCTAACTTGTGA-3’
hsa-miR-424-5p	5’-CAGCAGCAATTCATGTTTTGAA-3’
hsa-miR-19b-3p	5'-TGTGCAAATCCATGCAAAACTGA-3'
hsa-miR-146a-5p	5’-TGAGAACTGAATTCCATGGGTT-3’
hsa-miR-148b-3p	5’-TCAGTGCATCACAGAACTTTGT-3’
hsa-miR-1234-3p	5’-TCGGCCTGACCACCCACCCCAC-3’
hsa-miR-4454	5’-GGATCCGAGTCACGGCACCA-3’
hsa-miR-4284	5’-GGGCTCACATCACCCCAT-3’
hsa-miR-423-3p	5’-AGCTCGGTCTGAGGCCCCTCAGT-3’
hsa-miR-99a-5p	5’-AACCCGTAGATCCGATCTTGTG-3’
hsa-miR-494-3p	5’-TGAAACATACACGGGAAACCTC-3’
hsa-miR-21-5p	5’-TAGCTTATCAGACTGATGTTGA-3’
U6-Fw	5’-CGCTTCGGCAGCACATATACTA-3’
U6-Rev	5’-TTCACGAATTTGCGTGTCAT-3’
Universal-Rev	5’-GGATCGAGCACCAGTTACGC-3’
RNA5S1-Fw	5’-GGCCATACCACCCTGAAC-3’
RNA5S1-Rev	5’-GGTCTCCCATCCAAGTACTAAC-3’

Table S2. Experimentally validated target genes of differentially expressed miRNAs identified in HDM-treated versus control BEAS-2B cells based on miRTarBase v9.0.

**miR-99a-5p ↓**<br>AGO2<br>AKT1<br>CTDSPL<br>FGFR3<br>FKBP5<br>GRHL1<br>HOXA1<br>IGF1R<br>MTMR3<br>MTOR<br>PPP1CB<br>RAVER2<br>SERPINE1<br>SMARCA5<br>TRIB2<br><br>**miR-191-5p ↓**<br>BMP2<br>CCND2<br>CCR1<br>CDK6<br>CEBPB<br>CTDSP2<br>EGR1<br>IL1A<br>KRAS<br>LRRC8A<br>MDM4<br>NDST1<br>NUP50<br>SATB1<br>SLC16A2<br>SOX4<br>TET1<br>TMC7<br>YBX3<br><br>**miR-30e-3p ↓**<br>ATF3<br>NFKBIA	**miR-503-5p ↓**<br>ANLN<br>ARHGEF19<br>ATF6<br>BCL2<br>CCND1<br>CCND3<br>CCNE1<br>CCNE2<br>CCNF<br>CD40<br>CDC14A<br>CDC25A<br>CDKN1A<br>CHEK1<br>EIF2C1<br>FANCA<br>FBXW7<br>FGF2<br>FGF8<br>FGFR1<br>IGF1R<br>IKBKB<br>MIR503HG<br>PART1<br>PIK3R1<br>SMAD7<br>SMURF1<br>SMURF2<br>TNFRSF11A<br>VEGFA<br>WEE1<br><br>**miR-450a-5p ↓**<br>HNRNPK<br><br>**miR-361-5p ↓**<br>CXCR6<br>SND1<br>STAT6<br>VEGFA	**miR-181a-5p ↓**<br>ABCG2<br>AGO4<br>AHR<br>ATG5<br>ATM<br>BCL2<br>BCL2L11<br>C12ORF29<br>CARD11<br>CBLB<br>CCAT1<br>CCND1<br>CD4<br>CDH11<br>CDKN1B<br>CDX2<br>COL16A1<br>CTDSPL<br>DDIT4<br>DDX3X<br>DNMT3A<br>DUSP5<br>DUSP6<br>E2F5<br>EGFR<br>EREG<br>FOS<br>GATA6<br>GPD1L<br>GPR78<br>HIPK2<br>HRAS<br>IFNG<br>KAT2B<br>KLF6<br>KRAS<br>MAP2K1<br>MAPK1<br>MCL1<br>MEG3<br>MOSPD1<br>MTMR3<br>NLK<br>NOTCH1<br>OCLN<br>PBX3<br>PGR<br>PHACTR2<br>PHACTR4<br>PHLDA1<br>PHLPP2<br>PLAG1<br>POSTN<br>PPP3CA<br>PRAP1<br>PRKCD<br>PROX1<br>PTEN<br>PTPN11<br>PTPN22<br>RALA<br>RAP1B<br>RGS16<br>RGS5<br>RNF2<br>SASH1<br>SFRP4<br>SIRT1<br>SRGAP1<br>STAT3<br>TERT<br>TFAM<br>TGFBR1<br>TGFBRAP1<br>TIMP1<br>TUSC3<br>TWIST1<br>UBL3<br>WIF1<br>XIAP<br>ZNF763	**miR-148b-3p ↓**<br>ACVR1<br>ALCAM<br>CCKBR<br>CSF1<br>GADD45A<br>HLA-G<br>HMGA2<br>HOTAIR<br>HSP90AA1<br>ITGA5<br>MAP3K9<br>MLH1<br>NRAS<br>PIK3CA<br>PRKAB1<br>ROCK1<br><br>**miR-421 ↓**<br>ATM<br>CASP3<br>CBX7<br>CDH1<br>Circ_EXOC6B<br>EXOC6B<br>FGD5-AS1<br>FOXO4<br>RBMXL1<br>RSU1<br>SIRT3<br>SMAD4<br><br>**miR-151a-3p ↓**<br>CCNE1<br>NTRK3<br>TWIST1<br>ZNF763<br><br>**miR-361-3p ↓**<br>SH2B1<br>WNT10A	**miR-30a-3p ↓**<br>APBB2<br>ATF3<br>ATG12<br>CDK6<br>CRP<br>CYR61<br>DNMT3A<br>FMR1<br>HNRNPL<br>IL17A<br>KRT7<br>LMO7<br>MECP2<br>MYD88<br>SLC7A6<br>SNAI1<br>SOX9<br>THBS1<br>TMEM2<br>TUBA1A<br>TUT7<br>VEZT<br>WDR82<br>WNT2<br>XBP1<br><br>**miR-374a-5p ↓**<br>ATM<br>CEBPB<br>DICER1<br>FOXO1<br>GADD45A<br>MAGI2-AS3<br>MEG3<br>SRCIN1<br>WIF1<br>WNT5A	

**miR-25-3p ↓**	**miR-424-5p ↓**	**miR-15b-5p ↓**	**miR-93-5p ↓**	**miR-23a-3p ↓**	**miR-15a-5p ↓**
ATP2A2	AKT3	AMOT	CDH1	APAF1	AKT3
BCL2L11	ANLN	ATP2B2	CDKN1A	ARG1	APP
BTG2	APLN	AXIN2	DAB2	ATAT1	BACE1
CBR3-AS1	APLNR	BCL2	E2F1	BCL2	BCL2
CCL26	ATF6	CCND1	EREG	CDH1	BMI1
CDH1	BFAR	CCNE1	HIF1A	CELF1	BRCA1
CDKN1C	CADM1	COL12A1	HLA-F	CXCL12	CADM1
CPEB1	CCND1	CRIM1	ICAM1	FANCG	CARM1
DKK3	CCND3	EIF4A1	IL8	FAS	CCND1
DSC2	CCNE1	EMC4	ITGB8	FGF2	CCND2
ERBB2	CCNF	EPB41L4B	LAMC1	FOXO3	CCNE1
EZH2	CDC14A	FGF2	SMAD7	FOXP3	CDC25A
FBXW7	CDC25A	FOXO1	MYC	FZD5	CDCA4
HAND2	CDK6	FUT2	MYCN	G6PC	CHUK
KAT2B	CDX2	HNF1A	NPAS2	HAS2	CLCN3
KLF4	CHEK1	IFNG	MAPK9	HES1	CRKL
LATS1	CUL2	INSR	PTEN	HIP1R	CUL3
LATS2	FASN	KALRN	PURA	HMGB2	CXCL10
MAP2K4	FGF2	KDR	MSMO1	HMGN2	DLK1
MDM2	FGFR1	KRAS	SLC2A4	HOXB4	DMTF1
MS	HIF1A	MAGI2-AS3	TGFB1	IL6R	DRD1
MTF1	HOXA3	PPM1D	TGFBR2	IL8	FGF7
MYC	ITPR1	PURA	VEGFA	IRF1	FOXO1
NOX4	KDM5B	RECK	WNT2B	KLF3	GSDMB
PRMT5	KIF23	SMAD7	KAT2B	LAMP1	HMGA1
PTEN	LINC00324	SMURF1	TRIP10	LDHA	HMGA2
RECK	LINC00922	TMLHE	IGF2BP1	LDHB	HSPA1B
REV3L	MAP2K1	TRIM29	MAP3K2	LPAR1	IFNG
RUNX2	MYB	USP31	TUSC2	LRP5	KLF4
SMAD5	NFIA	VAMP1	MGLL	MEF2C	KLF6
SMAD7	PIAS1	VEGFA	SASH1	MT2A	MN1
TCEAL1	PLAG1		LATS2	MYH1	MYB
TGFBR2	PTCH1	**miR-362-5p**	SNX9	MYH2	NFKB1
TP53	SIAH1	CYLD	ATG16L1	MYH4	PDCD4
TWIST1	SMAD7	DSTN	ZBTB4	NEK6	PHF19
WDR4	SOCS2	NAP1L4	SNX16	POU4F2	PHLPP1
	SOCS6	NAP1L4P1	TP53INP1	PPARGC1A	PURA
**miR-362-3p ↓**	SPI1	SUZ12	SP7	PPP2R5E	RAB21
CASP9	WEE1		SLC16A9	PRKCA	RECK
CD82				PTEN	REPIN1
E2F1			**miR-660-5p ↓**	PTPN11	RET
ERG			SAA1	SIRT1	SKAP2
IL1B			SMARCA5	SLC25A15	SOX5
MAPK1			TFCP2	SPRY2	TMEM184B
MAPK3				TGFB2	TP53
MAPT				TMEM64	TSPYL2
ORM1				TNFAIP3	UCP2
PTPN1				TOP1	VEGFA
USF2				TRAF5	WNT3A
				XIAP	WT1
					YAP1

**miR-222-3p ↓**	TRPS1	**miR-34a-5p ↓**	HNF4A	SMAD4	
ADAM1A	VGLL4	ABLIM1	HOTAIR	SNAI1	
ARID1A	ZEB2	ACSL1	HPRT1	SNHG7	
BBC3	ZFAND5	ACSL4	IFNB1	SOX2	
BCL2L11		AGTR1	IL1B	SOX4	
C18orf25		AIM1	IL6R	SPI1	
CD47		AR	IMPA1	SRC	
CDKN1B		AREG	IMPDH2	STX1A	
CDKN1C		ARG1	INHBB	SYT1	
CERS2		ARHGDIB	JAG1	TCF7	
CORO1A		AXIN2	KCNH1	TGFB2	
DICER1		AXL	KDM4A	TGIF2	
DIRAS3		B4GALT5	KDR	TNF	
DIS3L2		BAX	KIT	TOM1	
DKK2		BCL2	KLB	TP53	
ESR1		BIRC5	KLF12	ULBP2	
ETS1		BMP7	KLF4	VAMP2	
FOS		CCL22	L1CAM	VEGFA	
FOXO3		CCND1	LDHA	WNT1	
GAS5		CCND3	LEF1	XBP1	
GJA1		CCNE2	MAGEA12	YY1	
GNAI2		CD24	MAGEA2	ZAP70	
GNAI3		CD44	MAGEA3		
ICAM1		CDC25A	MAGEA6		
INPP4B		CDK4	MAP2K1		
KIAA1370		CDK6	MAP3K9		
KIT		CDKN2A	MARCH5		
LYPLA1		CDKN2C	MDM4		
MEGF9		CEBPB	MET		
MGMT		CLOCK	MMP2		
MIAT		CSF1R	MMP7		
MMP1		CTNNB1	MTA2		
PDCD10		CXCL10	MYB		
PHACTR4		CXCL11	MYC		
PPP2R2A		CXCR3	MYCN		
PRDM1		CYBB	NAMPT		
PTEN		DGUOK	NANOG		
RECK		DLL1	NCOA1		
SELE		DOCK3	NOTCH1		
SOCS1		E2F1	NOTCH2		
SOD2		E2F3	NUMB		
SSSCA1		EPHA5	PCBP2		
SSX2IP		FOSL1	PDGFRA		
STAT5A		FOXP1	PDGFRB		
TCEAL1		FOXP3	PEA15		
TIMP2		FUT8	POU5F1		
TIMP3		GALNT7	PPARA		
TIPARP		GAS1	PPP1R10		
TMED7		GFRA3	RICTOR		
TNFSF10		GNAI2	RNF169		
TP53		GRM7	SIRT1		
TP53BP2		HDAC1	SIRT7		

**miR-1915-3p** ↑	**miR-19b-3p** ↑	**miR-126-3p**↑ VEGFA	**miR-106b-5p** ↑	**miR-19a-3p** ↑
BCL2	AQP5	ADAM9	APC	ABCA1
	ARID4B	ADM	APP	AKT1
**miR-1234-3p** ↑	ATXN1	AKT1	ATG16L1	ARIH2
STAT3	BACE1	ANXA2	BCL2L11	ATXN1
	BCL2L11	BCL2	CASP7	BCL2L11
**miR-423-5p** ↑	BMPR2	BMP2	CASP8	BMPR2
ADCY3	CCL1	CADM1	CCND1	CCNA2
CASC9	CIT	CAMSAP1	CCND2	CCND1
CXCL12	CLOCK	CCNE2	CDKN1A	CD22
FAM3A	CREB1	CD97	CNN1	CUL5
LOC105371414	CUL5	Cdkn1b	CRY2	DNMT1
PLCB1	CYP19A1	CRK	DAB2	DPYSL2
SOX12	DNMT1	CRKL	E2F1	ERBB4
TFF1	ESR1	CXCL12	E2F2	ESR1
TNIP2	F8	CXCR4	E2F3	FBXO32
USF2	FBXO32	DNMT1	E2F5	HIPK2
	GCM1	E2F1	EOMES	HOXA5
**miR-423-3p** ↑	HIPK1	EGFL7	FAM129A	IL6
CDKN1A	HIPK3	FOXO3	FAS	IMPDH1
HDAC5	HOXA9	HOXA9	FYN	KAT2B
RUNX1	IGF1	IGFBP2	HIF1A	KIT
STAT1	KAT2B	IRS1	IRF1	MECP2
	MTOR	JAK1	ITCH	MSMO1
**miR-590-5p** ↑	MXD1	KLF10	JAK1	MXD1
CHL1	MYCN	KRAS	KAT2B	MYC
CREB5	MYLIP	LNPK	MAPK9	MYCN
FTX	NCOA3	LRP6	MFN2	NPEPL1
RBPJ	PPP2R5E	MERTK	MMP2	NR4A2
STAT3	PRKAA1	MMP7	MXI1	PHLPP1
TGFBR2	PTEN	NFKBIA	MYC	PKNOX1
	SMAD4	PARP16	MYCN	PRMT5
	SOCS1	PGR	PKD2	PSAP
	SOCS3	PIK3CG	PRRX1	PTEN
	STAT3	PIK3R2	PTEN	RAB13
	TGFB1	PITPNC1	PTENP1	RAB14
	TGFBR2	PLK2	PURA	SMAD4
	TLR2	PLK4	RB1	SOCS1
	TNFRSF12A	PTPN7	RBL1	SOCS3
	TP53	RGS3	RBL2	SUZ12
	TRIM54	RHOU	RUNX3	TF
		SIRT1	SMAD7	TGFBR2
		SLC45A3	SNHG16	TLR2
		SLC7A5	STAT3	TNF
		SOX2	TCEAL1	TRIM54
		SPRED1	TCF4	VPS4B
		TCF4	TP53	ZBTB4
		TEK	TWIST1	
		TOM1	VEGFA	
		TWF1	WEE1	
		TWF2	ZBTB4	
		VCAM1		

**miR-31-5p** ↑	SP7	**miR-146a-5p** ↑	NOTCH2	**miR-24-3p** ↑	LDHA
ACOX1	SRC	ADIPOQ	NUMB	ACVR1B	LDHB
ABCB9	STK40	BCLAF1	PA2G4	AGPAT2	MAFB
AGO3	TBXA2R	BGLAP	PRKCE	ANGPT2	MAP3K9
APP	TIAM1	BRCA1	PTGES2	ARHGAP19	MAPK14
ARID1A	WASF3	BRCA2	PTGS2	AURKB	MCM10
ARPC5	YY1	CARD10	RAC1	BCL2L11	MCM4
BACE1		CASP7	RARB	BCL2L12	MEN1
C11orf30		CCDC6	RHPN2	BRCA1	MLEC
CDK1		CCL5	RNF11	CARD10	MMP14
CREG1		CCNA2	ROBO1	CCNA2	MRTFA
CXCL12		CD40LG	ROCK1	CCND1	MXI1
DACT3		CDKN1A	S100A12	CD34	MYC
DICER1		CDKN3	SIKE1	CDK1	NCAN
DKK1		CFH	SLPI	CDK4	NCSTN
DMD		CNOT6L	SMAD2	CDKN1B	NDST1
E2F2		COPS8	SMAD4	CDKN2A	NFAT5
ETS1		COX2	SMN1	CDX2	NOS3
FOXP3		CPM	SOS1	CHEK1	NOTCH1
FZD3		CTGF	SOX2	CNDP2	PAK4
GAS1		CXCL12	SPP1	COPS5	PCNA
HIF1A		CXCR4	STAT1	CORO1A	PER2
HIF1AN		DUSP1	TAB2	CTSD	PIK3R3
HOXC13		EGFR	TFDP2	CYP11B2	POLD1
ICAM1		ELAVL1	TIGAR	DEDD	PRKCH
ITGA5		ERBB4	TLR2	DHFR	PSAP
JAZF1		FADD	TLR4	DHFRP1	PTPN9
KLF13		FAF1	TRAF6	DND1	PTPRF
LATS2		FAS	UHRF1	DYRK2	REG4
MAP4K4		GLS	WASF2	E2F1	RRM2
MCM2		GLS2	ZNF117	E2F2	S100A8
MET		HNRNPD		E2F3	SH3PXD2A
MLH1		HOXD10		EIF2S3	SP1
MMP16		ICAM1		FAF1	SSSCA1
MMP3		IL1RAP		FEN1	ST7L
MPRIP		IL1RL2		FGFR3	STX16
NFAT5		IL6		FURIN	TACC3
NUMB		IL8		H2AFX	TG
PPP2R2A		IRAK1		HMOX1	TGFB1
PRKCE		IRAK2		HNF4A	TMED7
RAB27A		IRAK4		HPCA	TOP1
RASA1		KIF22		IFNG	TRIB3
RDX		L1CAM		IFNR	UBC
RET		LAMC2		IGF1	UBD
RHOA		MALAT1		INS	WNT4
RHOBTB1		MTA2		INSIG1	XIAP
SATB2		MYD88		JPH2	ZNF217
SELE		MYO6		KHSRP	ZNF317
SLC20A1		NFKB1		LAMB3	

Table S3. Experimentally validated target genes of differentially expressed miRNAs identified in HDM-treated versus control BEAS-2B-derived EVs based on miRTarBase v9.0.

**miR-494-3p ↓**	**miR-21-5p ↓**	ICAM1	RTN4
ARHGAP5	AKT2	IGF1R	SASH1
ARNTL	AMH	IL12A	SATB1
ATF3	ANKRD46	IL1B	SECISBP2L
BAG1	ANP32A	INTU	SERPINB5
BCL2	AP1AR	IRAK1	SERPINI1
BCL2L11	APAF1	ISCU	SETD2
BMI1	BASP1	JAG1	SIRT2
CDK6	BCL2	JMY	SMAD7
CFTR	BCL6	KIF27	SMARCA4
CNR1	BMPR2	LATS2	SMN1
CXCR4	BTG2	LRRFIP1	SOD3
FOXJ3	CASC2	MAP2K3	SOX17
HGAC2	CBX4	MARCKS	SOX2
HNRNPA3	CCL20	MEF2C	SOX5
HOXA10	CCR1	MKNK2	SOX6
ITPR1	CCR7	MMP2	SP1
MAP2K1	CDC25A	MMP9	SPRY1
MCC	CDIP1	MSH2	SPRY2
MYC	CDK2AP1	MSH6	STAT3
PDIA3	CDK6	MTAP	STK36
PROS1	CLU	MYC	STRN
PTEN	COL4A1	MYD88	TCF21
RAD23B	CXCL10	NCAPG	TGFBI
SDC1	DAXX	NCOA3	TGFBR2
SLC26A3	DDAH1	NFIA	TGFBR3
SYNCRIP	DERL1	NFIB	TGIF1
TEAD1	DNM1L	NTF3	TIAM1
TFAM	DOCK4	PACRG	TIMP3
TGFB1	DOCK5	PCBP1	TLR2
ZEB1	DOCK7	PDCD4	TM9SF3
	DUSP10	Pdcd4	TNFAIP3
	E2F1	PELI1	TNFRSF10B
	E2F2	PIAS3	TOPORS
	EGFR	PLAT	TOR1AIP2
	EIF4A2	PLOD3	TP53BP2
	ELAVL4	PPARA	TP63
	ENO4	PPIF	TPM1
	ERBB2	PTEN	VEGFA
	ETNK1	PTX3	VHL
	FASLG	RASA1	WWC2
	FMOD	RASGRP1	WWP1
	GAS5	RECK	YOD1
	GDF5	REST	ZBTB7A
	GLS	RFFL	
	GLS2	RHO	
	HIPK3	RHOB	
	HNRNPK	RMND5A	
	HPGD	RPS7	

**miR-612 ↑**<br>AKT2<br>BAX<br>CASP3<br>FOXK1<br>RAP1B<br>TP53<br><br>**miR-302d-3p ↑**<br>AKT1<br>ARL4C<br>CCND2<br>CDK2<br>ERBB4<br>IL8<br>KLF13<br>LEFTY1<br>LEFTY2<br>MAP3K1<br>MBNL2<br>NR4A2<br>TRPS1<br>VEGFA<br><br>**miR-873-3p ↑**<br>UMAD1<br>ZIC2<br><br>**miR-1246 ↑**<br>AR<br>CCNG2<br>CFTR<br>DNAH3<br>DYRK1A<br>ELAVL1<br>GLS2<br>MIF<br>NFIB<br>SPRED2<br>SRSF1	**miR-15a-5p ↑**<br>AKT3<br>APP<br>BACE1<br>BCL2<br>BMI1<br>BRCA1<br>CADM1<br>CARM1<br>CCND1<br>CCND2<br>CCNE1<br>CDC25A<br>CDCA4<br>CHUK<br>CLCN3<br>CRKL<br>CUL3<br>CXCL10<br>DLK1<br>DMTF1<br>DRD1<br>FGF7<br>FOXO1<br>GSDMB<br>HMGA1<br>HMGA2<br>HSPA1B<br>IFNG<br>KLF4<br>KLF6<br>MN1<br>MYB<br>NFKB1<br>PDCD4<br>PHF19<br>PHLPP1<br>PURA<br>RAB21<br>RECK<br>REPIN1<br>RET<br>SKAP2<br>SOX5<br>TMEM184B<br>TP53<br>TSPYL2<br>UCP2<br>VEGFA<br>WNT3A<br>WT1<br>YAP1	**miR-125b-5p ↑**<br>ABTB1<br>ACADS<br>AHRR<br>AKT1<br>ARID3A<br>ARID3B<br>BAK1<br>BAX<br>BBC3<br>BCL2<br>BCL2L2<br>BCL3<br>BMF<br>BMPR1B<br>BTG2<br>CBFB<br>CCNB1<br>CCNJ<br>CD4<br>CDH5<br>CDK1<br>CDKN2A<br>CPSF6<br>CSNK2A1<br>CYP24A1<br>DGAT1<br>DKK3<br>DRAM2<br>DUSP6<br>E2F2<br>E2F3<br>EIF4EBP1<br>EIF5A2<br>ENPEP<br>EPO<br>EPOR<br>ERBB2<br>ERBB3<br>ETS1<br>FGFR2<br>FOXA1<br>GLI1<br>GPC1<br>GRIN2A<br>GSS<br>HK2<br>HMGA1<br>HMGA2<br>ICAM2<br>IGF2<br>IKZF2<br>IKZF3<br>IKZF4<br>IL6R<br>IRF4<br>JUB<br>JUN<br>JUND<br>KCNA1<br>KLF13<br>KRAS<br>KRT7<br>LACTB<br>LDHA<br>LIF<br>LIN28A<br>LIN28B<br>LIPA<br>MAN1B1<br>MAP2K7<br>MAP3K11<br>MAPK14<br>MCL1<br>MEGF9<br>MMP13<br>MMP2<br>MMP26<br>MUC1<br>MX1<br>MXD1<br>NCOR2<br>NES<br>NKIRAS2<br>NTRK3<br>PCTP<br>PIAS3<br>PIGF<br>PIK3CD<br>PP1CAB<br>PPP1CA<br>PRDM1<br>PRKRA<br>PRTG<br>RAF1<br>RPS6KA1<br>SCNN1A<br>SEMA4D<br>SET<br>SFRP5<br>SGPL1<br>SIRT7<br>SMAD4<br>SMO<br>SMYD2<br>SNAI1<br>SOX11<br>SPHK1<br>STARD13<br>STAT3<br>SUV39H1<br>TBC1D1<br>TEF<br>TET2<br>TNF<br>TNFAIP3<br>TP53<br>TP53INP1<br>TRAF6<br>TRIAP1<br>TRIM71<br>VDR<br>VPS4B<br>VPS51<br>4632428N05Rik

Table S4. Asthma relevance of Figure S9 target genes differentially expressed in severe asthma compared with healthy controls.

Gene (Severe asthma vs. Healthy control)	Reported relevance to asthma and airway biology	Interpretation relative to present study
**BAX ↑**	Pro-apoptotic regulator involved in epithelial-cell apoptosis and resolution of mucous-cell hyperplasia. Reduced BAX expression has previously been reported in asthmatic airway epithelium and following IL-13 stimulation.	Asthma relevant; expression direction differs from prior reports.
**MCL1 ↑**	Anti-apoptotic protein that promotes eosinophil survival. MCL1 overexpression exacerbates eosinophilic airway inflammation and mucus production in experimental asthma.	Supports enhanced pro-survival inflammatory signaling.
**RUNX2 ↑**	IL-13 and HDM induce RUNX2 expression in airway epithelium. RUNX2 promotes SPDEF-dependent goblet-cell differentiation and mucus production. Increased epithelial RUNX2 has been reported in asthma.	Strong concordance with established asthma-associated epithelial remodeling.
**STAT3 ↓**	STAT3 activation contributes to allergic airway inflammation and epithelial responses to IL-13. Functional activity is often more relevant than transcript abundance.	Functionally relevant, although transcript-level changes are difficult to interpret.
**AKT2 ↓**	Participates in airway epithelial inflammatory signaling through NF-κB and cytokine regulation.	Supports involvement of epithelial inflammatory pathways.
**BMAL1/ ARNTL ↑**	Core circadian-clock regulator expressed in airway epithelium. Altered circadian rhythmicity has been associated with asthma.	Suggests circadian-clock dysregulation in severe asthma.
**HMGA2 ↓**	Downstream mediator of TGF-β-induced epithelial-mesenchymal transition (EMT) and remodeling programs.	Consistent with remodeling-related pathways, although direct asthma expression evidence is limited.
**IRAK1 ↑**	Mediator of NF-κB signaling and target of miR-146a in airway epithelial cells. Previous studies reported minimal differences in IRAK1 expression between asthma and controls.	Indicates inflammatory pathway activation; may reflect disease- or cohort-specific regulation.
**SNAI1 ↓**	Master regulator of EMT induced by TGF-β and allergen exposure. Increased expression has been reported in asthmatic bronchial mucosa.	Mechanistically relevant, but expression direction differs from published observations.
**WNT3A ↓**	Component of WNT/β-catenin signaling implicated in airway remodeling and EMT. Direct differential expression in asthma remains inconsistent.	Supports remodeling-associated signaling pathways.
**TLR2 ↑**	Increased TLR2 expression has been reported in airway epithelium from asthma patients, including severe disease.	Strong concordance with previous human asthma studies.

Gene (Severe asthma vs. Healthy control)	Reported relevance to asthma and airway biology	Interpretation relative to present study
**YAP1 ↓**	Hippo/YAP signaling contributes to airway remodeling and epithelial responses, although findings appear cell-type specific.	Supports remodeling biology despite differing directional changes among cell populations.
**ZEB1 ↑**	EMT-associated transcription factor involved in TGF-β-driven airway remodeling.	Consistent with enhanced EMT and remodeling processes.
**ZEB2 ↑**	Mediates EMT in airway epithelial cells and has been reported to be elevated in eosinophilic asthma, correlating with remodeling and inflammatory markers.	Strong concordance with severe asthma-associated remodeling.
**BCL2 ↓**	Anti-apoptotic regulator implicated in altered epithelial BCL2/BAX balance in asthma. Increased BCL2 has been reported in allergic asthma.	Asthma relevant, though expression direction differs from prior studies.
**CCND2 ↓**	Cell-cycle regulator associated with airway smooth-muscle proliferation and remodeling pathways.	Supports remodeling-related mechanisms but has limited epithelial-specific evidence.
**MYD88 ↑**	Central adaptor for Toll-like receptor signaling and a key regulator of allergen-induced immune responses.	Consistent with activation of innate immune signaling pathways.
**SMAD4 ↓**	Essential mediator of TGF-β signaling implicated in airway inflammation, remodeling, and epithelial responses.	Consistent with altered TGF-β pathway regulation, although prior model systems reported increased expression.
**TGFBR2 ↑**	TGF-β receptor involved in epithelial barrier dysfunction, EMT, and airway fibrosis/remodeling.	Strong concordance with activation of a pro-remodeling TGF-β axis.

Several differentially expressed Fig. S9 target genes have established roles in airway epithelial inflammation, innate immune signaling, apoptosis, circadian regulation, and epithelial-mesenchymal transition, including RUNX2, TLR2, ZEB1, ZEB2, MYD88, and TGFBR2, providing independent biological support for the remodeling and inflammatory pathways identified in severe asthma.
